# Nanomedicines Bearing an Alkylating Cytostatic Drug from the Group of 1,3,5-Triazine Derivatives: Development and Characterization

**DOI:** 10.3390/pharmaceutics14112506

**Published:** 2022-11-18

**Authors:** Ekaterina Sinitsyna, Irina Bagaeva, Erik Gandalipov, Evgenia Fedotova, Viktor Korzhikov-Vlakh, Tatiana Tennikova, Evgenia Korzhikova-Vlakh

**Affiliations:** 1 Institute of Macromolecular Compounds, Russian Academy of Sciences, Bolshoy pr. 31, St. Petersburg 199004, Russia; 2 Institute of Chemistry, Saint-Petersburg State University, Universitetsky pr. 26, St. Petersburg 198504, Russia; 3 International Institute of Solution Chemistry and Advanced Materials Technologies, ITMO University, Lomonosov Street 9, St. Petersburg 191002, Russia

**Keywords:** 1,3,5-triazine derivatives, dioxadet, nanomedicines, anticancer drugs, polymer nanoparticles, nanoprecipitation, PEG-copolymers, poly(lactic acid), poly(ε-caprolactone)

## Abstract

Cancer is still one of the major diseases worldwide. The discovery of new drugs and the improvement of existing ones is one of the areas of priority in the fight against cancer. Dioxadet ([5-[[4,6-bis(aziridin-1-yl)-1,3,5-triazin-2-yl]amino]-2,2-dimethyl-1,3-dioxan-5-yl]methanol) represents one of the promising 1,3,5-triazine derivatives and has cytostatic activity towards ovarian cancer. In this study, we first report the development of dioxadet-bearing nanomedicines based on block-copolymers of poly(ethylene glycol) monomethyl ether (mPEG) and poly(D,L-lactic acid) (PLA)/poly(ε-caprolactone) (PCL) and then conduct an investigation into their characteristics and properties. The preparation of narrow-sized nanoparticles with a hydrodynamic diameter of 100–120 nm was optimized using a nanoprecipitation approach. Thoughtful optimization of the preparation of nanomedicines was carried out through adjustments to the polymer’s molecular weight, the pH of the aqueous medium used for nanoprecipitation, the initial drug amount in respect to the polymer, and polymer concentration in the organic phase. Under optimized conditions, spherical-shaped nanomedicines with a hydrodynamic diameter of up to 230 nm (PDI < 0.2) containing up to 592 ± 22 μg of dioxadet per mg of polymer nanoparticles were prepared. Study of the drug’s release in a model medium revealed the release up to 64% and 46% of the drug after 8 days for mPEG-*b*-PLA and mPEG-*b*-PCL, respectively. Deep analysis of the release mechanisms was carried out with the use of a number of mathematical models. The developed nanoparticles were non-toxic towards both normal (CHO-K1) and cancer (A2780 and SK-OV-3) ovarian cells. A cell cycle study revealed lesser toxicity of nanomedicines towards normal cells and increased toxicity towards cancer cells. The IC_50_ values determined for dioxadet nanoformulations were in the range of 0.47–4.98 μg/mL for cancer cells, which is close to the free drug’s efficacy (2.60–4.14 μg/mL). The highest cytotoxic effect was found for dioxadet loaded to mPEG-*b*-PCL nanoparticles.

## 1. Introduction

To date, 1,3,5-triazine derivatives have been extensively studied as antimicrobial [1,2], antiviral [3,4], anti-inflammatory [5,6], and anticancer [7,8] drugs. 1,3,5-Triazine derivatives are symmetrical triazine compounds, also abbreviated as s-triazines, and represent one of three possible isomers of a six-membered ring containing three nitrogen atoms alternating with carbon atoms. Many of the s-triazines exhibit anticancer activity related to their alkylating properties [9]. In particular, many s-triazines are capable of damaging the DNA of cancer cells by alkylating DNA purine bases. Such s-triazines are used to treat several cancers [9,10,11,12].

**Figure 1:**
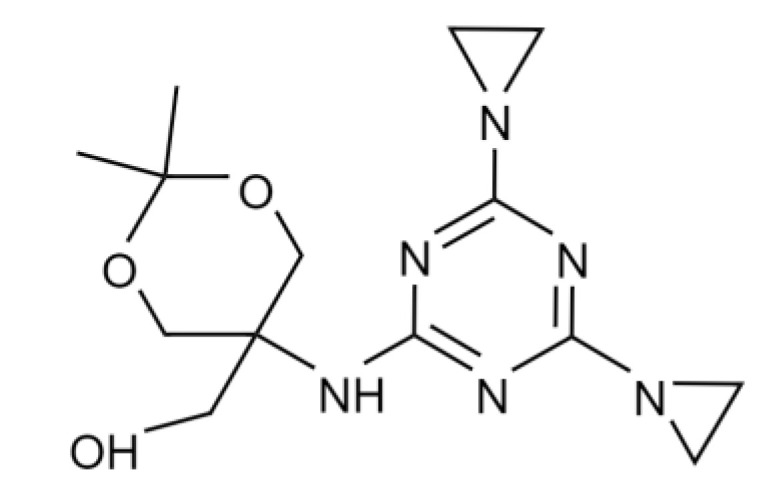
Chemical structure of [5-[[4,6-bis(aziridin-1-yl)-1,3,5-triazin-2-yl]amino]-2,2-dimethyl-1,3-dioxan-5-yl]methanol (dioxadet, DOD).

One of the most prospective 1,3,5-triazine derivatives is [5-[[4,6-bis(aziridin-1-yl)-1,3,5-triazin-2-yl]amino]-2,2-dimethyl-1,3-dioxan-5-yl]methanol, which is also known as dioxadet (DOD) (Figure 1). This substance is amphiphilic and can be administrated in oil or in aqueous solutions [13]. DOD is strongly cytotoxic against ovarian cancer cells, but its local and systemic side effects are much less than those of platinum-based anti-tumor drugs [10,14].

Recently, Mikolaichuk et al. presented the results of a comprehensive study of the cytotoxic, hemolytic, and antioxidant properties of DOD [15]. The authors revealed high hemocompatibility with low affinity to HSA. At the same time, DOD reduced the viability of several cancer cell lines (A-549, PA-1, T98G, SK-HEP-1, and PANC-1) in a dose-dependent manner. Among the studied cells, the greatest effect was detected for the A-549 line. DNA damage and inhibition of reactive oxygen species production have been proposed by the authors as two key pathways of the cytotoxic action of DOD.

Based on preclinical studies, myelotoxicity was found to be the key dose-limiting side effect of DOD, while other side effects were not pronounced. In comparison, cisplatin and other platinum drugs, even when administered intraperitoneally, have hematotoxic, ototoxic, neurotoxic, and nephrotoxic effects [16,17,18,19]. Experiments in rats with ovarian cancer have shown an increase in the lifespan of animals of 63.1% after treatment with DOD, while for the administration of cisplatin this parameter was 48.1%. According to the results of a clinical study, DOD was found to be highly effective against stage III-IV ovarian tumors when administered intravenously or intraperitoneally in a single dose of 15 mg at 72–96 h intervals up to a total dose of 90–120 mg. Moreover, the therapeutic efficiency of DOD was even demonstrated in patients who had previously received unsuccessful treatment with other alkylating cytostatic drugs [20]. 

However, as with many other alkylating agents, 1,3,5-triazins are toxic to normal cells and also can be cancerogenic [21,22]. These drawbacks can be overcome by developing encapsulated forms of s-triazines that reduce acute systemic toxicity and improve bioavailability via localization of a drug within the tumor, as well as provide its prolonged release. The introduction of various nanoparticles into biomedicine has become increasingly popular due to their ability to deliver various cargoes to a specific biological target [23,24]. Currently, many nanotechnology-based drug delivery systems have been approved for clinical use (Genexol-PM^®^, Myocet^®^, Polyglumex^®^, etc.), and many systems are in various stages of preclinical and clinical trials [25]. As for DOD, the development of DOD nanogels based on a non-biodegradable block copolymer of poly(ethylene glycol) and poly(methacrylic acid) cross-linked with ethylenediamine or cysteamine was reported by Voeikov et al. [26]. The resulting nanoformulations had a diameter of 130 nm and were narrowly dispersed (PDI = 0.09). The half-minimum inhibitory concentration (IC_50_) values determined for those nanoformulations were in the range of 204–564 μg/mL, depending on the cross-linking of the polymer and the glioma cell line (C6 or U87). The IC_50_ values for free DOD were 34 µg/mL for U87 cells and 214 µg/mL for C6 cells. 

In this study, we focused on the development of DOD nanoformulations based on block-copolymers of poly(ethylene glycol) monomethyl ether (mPEG) with biodegradable poly(lactic acid) (PLA)/poly(ε-caprolactone) (PCL). All selected polymers have been approved by the United States Federal Agency on Food and Drug Administration (FDA) as biomedical polymers [27,28]. Currently, there are a number of anticancer nanopharmaceutics based on PEG-*b*-PLA or PLA/PEG-based copolymers that are commercially available or involved in clinical trials [25]. For instance, a paclitaxel nanoformulation based on mPEG-*b*-PLA has been approved under the Genexol-PM trade name for the treatment of breast cancer. A BIND-014 nanoformulation, representing docetaxel encapsulated into nanoparticles based on block-copolymers of PEG with poly(lactic acid-*co*-glycolic acid), is undergoing phase II clinical trials for the treatment of non-small-cell lung cancer. 

**Figure 2:**
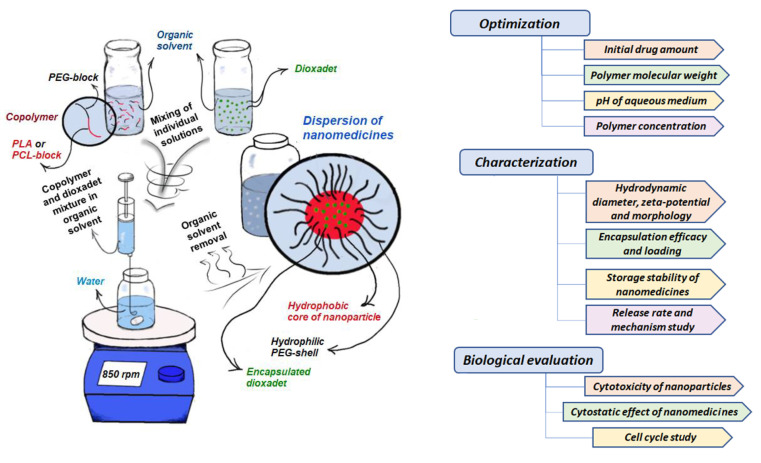
Scheme of the encapsulation of dioxadet into mPEG-*b*-PLA/mPEG-*b*-PCL nanoparticles and the design of the study.

In order to prepare the encapsulated forms of DOD, the drug loading technique was optimized to obtain narrowly dispersed nanoparticles. The effect of polymer composition and molecular weight, medium, the initial amount of the drug, and preparation modes on the nanoparticles’ characteristics and drug loading were comprehensively studied. The obtained nanoformulations were investigated in terms of their stability, rate, and mechanism of drug release. Finally, the effect of DOD-loaded nanoparticles on the viability and proliferation of normal ovarian cells and ovarian cancer cells was examined and compared to the effect of free DOD. The scheme illustrating DOD encapsulation and the design of the experimental work are presented in Figure 2.

## 2. Materials and Methods

### 2.1. Materials 

D,L-lactide, poly(ethylene glycol) monomethyl ether (mPEG, *Mn* = 5000), and 2-ethylhexanoate tin (II) (tin octoate, Sn(Oct)_2_) were purchased from Sigma-Aldrich (Darmstadt, Germany). ε-caprolactone was procured from J&K Scientific (Shanghai, China). Dioxadet (2,2-Dimethyl-5-(4,6-diaziridinyl-2-*s*-triazinylamino)-*m*-dioxane-5-methanol, 99%) was purchased from Chemconsult (St. Petersburg, Russia). D,L-Lactide was recrystallized from toluene and dried before polymerization overnight under vacuum at 30 °C. 

Solvents: toluene, hexane, chloroform, methanol, methylene chloride, dodecanol, and tetrahydrofuran (THF) were obtained from Vecton (St. Petersburg, Russia) and purified using standard protocols. Acetonitrile (ACN) was purchased from J.T. Baker (Phillipsburg, NJ, USA) and used without additional purification. 

Dialysis membranes with molecular weight cut-offs (MWCO) of 2000 and 100,000 were obtained from Orange Scientific (Braine-l’Alleud, Belgium); Spectra/POR^®^ Biotech CE with an MWCO of 300,000 was purchased from Serva (Heidelberg, Germany). Hydrophobic PTFE syringe filters with a nozzle diameter of 13 mm and pore size of 0.45 µm were obtained from Millex Merck-Millipore (Burlington, MA, USA). 

Human ovarian adenocarcinoma cell lines (A2780, SK-OV-3) and Chinese hamster ovarian cells (CHO-K1) were purchased from Blokhin Cancer Institute’s collection (Moscow, Russia). According to the manufacturer’s recommendations, cells were cultivated in RPMI-1640, DMEM, and DMEM/F12 (Biolot, St. Petersburg, Russia). All complete media contained 10% FBS (HyClone, Logan, UT, USA) and 50 µg/mL gentamicin (Biolot, St. Petersburg, Russia). MTT (Sigma Aldrich, St. Louis, MO, USA) stock solution was prepared by dissolving MTT powder in PBS to a concentration of 5 mg/mL, followed by filtration through a 0.22 nm PES filter.

### 2.2. Instruments 

The introduction of the organic phase into the aqueous phase was carried out using an Instilar 1438 Dixion infusion pump (Moscow, Russia). Magnetic stirrers and a Unimax 1010 thermoshaker from Heidolph (Schwabach, Germany) were used for mixing. A VaCo 5–II lyophilizer Zirbus (Bad Grund, Germany) was used to dry the obtained samples. The hydrodynamic radius and ζ-potential of the particles were analyzed (at a scattering angle of 173 °C) using a Zetasizer Nano ZS Malvern Panalytical particle analyzer (Malvern, UK) and a NanoSight NS300 particle analyzer (Malvern, UK). UV absorption measurements were carried out on a UV–1800 Shimadzu spectrophotometer (Kyoto, Japan). SEC analyses were performed on a Shimadzu LC-20 Prominence chromatograph with an RID 10-A refractometric detector (Kyoto, Japan) equipped with a tandem of two Agilent PLgel MIXED-D columns (5 µm, 7.5 mm × 300 mm, Agilent Technologies, Santa-Clara, CA, USA). NMR spectroscopy was carried out using a Bruker Avance III WB at 400 MHz (Karlsruhe, Germany).

### 2.3. Methods

#### 2.3.1. Synthesis of mPEG-*b*-PLA and PLA

Both mPEG-*b*-PLA and PLA were synthesized by ring-opening polymerization of D,L- lactide (D,L-LD) in bulk (Figure S1, Supplementary Materials). mPEG monomethyl ether (*Mn* = 5000) was used as a macroinitiator in the synthesis of PEGylated copolymer. D,L-lactide was recrystallized from toluene. The purified monomer was dried before polymerization overnight under vacuum at 30 °C. To obtain mPEG-*b*-PLA or PLA, 5 g of D,L-lactide was taken for the reaction. PLA was synthesized as described earlier [29]. In the case of copolymer, the following ratios were used: [D,L-lactide]:[St(Oct)_2_] = 1000, [D,L-lactide]:[mPEG] = 1500. The reagents were placed into a Schlenk tube equipped with a magnetic stirrer. The remaining solvent was removed using a vacuum pump, 2 mL of hexane was added, and the solvent was pumped out again using a vacuum line. Subsequently, 0.3 wt% St(Oct)_2_ from the mass of monomers was introduced into the reaction mixture. Polymerization was carried out under vacuum in an oil bath at 130 or 140 °C. The polymerization time was 60 or 90 min. The resulting polymer was dissolved in chloroform. The obtained solution was concentrated using a rotary evaporator and precipitated in a twenty-fold volume of cold methanol. The resulting polymer product was dried in a vacuum desiccator for 24 h. The polymer yield varied in the range of 60 to 90% depending on the conditions. ^1^H NMR for mPEG-*b*-PLA (CHCl_3_, 25 °C) (δ, ppm): PLA: 1.6 (-CH_3_), 5.15–5.20 (-CH); PEG: 3.67 (-CH_2_) (Figure S2, Supplementary Materials). The obtained polymers were characterized by size-exclusion chromatography (SEC) to determine weight-average molecular weight (*M_w_*) and dispersity (*Đ*). THF was used as the mobile phase in SEC analysis. The eluent flow rate was 1.0 mL/min and the analysis temperature was 40 °C. Molecular-weight characteristics were calculated using the calibration plot built for standard polystyrene samples with molecular weights ranging from 2000 to 450,000 (Agilent Technologies, Santa Clara, CA, USA and Waters, Milford, MA, USA). 

#### 2.3.2. Synthesis of mPEG-*b*-PCL and PCL

Both mPEG-*b*-PCL and PCL were synthesized by ring-opening polymerization of ε-caprolactone in bulk (Figure S1, Supplementary Materials). A total of 10 mL of ε-caprolactone was placed into a 50 mL round-bottomed flask equipped with a magnetic stirrer and tin octoate (II) and a co-initiator was added. Various ratios ([ε-caprolactone]:[St(Oct)_2_] = 1000, [co-initiator]:[tin octoate] = 1.9–5.2) were used to select optimal conditions. Dodecanol and mPEG acted as co-initiators. Polymerization was carried out by heating in an oil bath at 140 °C with intensive stirring. The polymerization time was varied from 4 to 24 h. After the reaction was completed, the polymer product was dissolved in chloroform and precipitated in a 10-fold volume of cold methanol. The resulting polymer was left to dry in a vacuum desiccator for 24 h. The polymer yield varied from 75 to 93% depending on the polymerization conditions. ^1^H NMR for mPEG-*b*-PCL (CHCl_3_, 25 °C) (δ, ppm): PCL: 1.4−1.6 (-CH_2_-), 2.3 (-CH_2_CO-), 4.2 (-OCH_2_); PEG: 3.67 (-CH_2_) (Figure S3, Supplementary Materials). The obtained polymers were characterized by SEC to determine *M_w_* and *Đ* as described in Section 2.3.1.

#### 2.3.3. Preparation of Nanoparticles by Nanoprecipitation 

The nanoprecipitation technique was used to prepare nanoparticles with an average diameter around 100 nm. A stable suspension of monodisperse polymer nanoparticles can be obtained in a narrow metastable area for a polymer/organic solvent/water mixture. This region is located between the binodal and spinodal curves in the three-component phase diagram and corresponds to dilute polymer solutions and a large amount of water (Figure S4, Supplementary Materials). A total of 20 mg of polymer was dissolved in 4 mL of ACN, ACN/THF = 1/1 (*v*/*v*), or THF (polymer concentration 5 mg/mL) and left to mix at 120 rpm at 30 °C for 24 h. Using a pump, an organic solution was injected into water at a flow rate of 2 mL/min under vigorous stirring (850 rpm). The ratio of the organic phase to the aqueous one was 1:5 (*v*/*v*). To remove the organic solvent, the system was left open while being stirred at 22 °C for 24 h. The nanoparticle yield was in the range of 60 to 90%. The resulting polymer nanoparticles were stored at 4 °C. 

The average hydrodynamic diameter (*D_H_*), polydispersity index (PDI), and ζ-potential of the obtained nanoparticles in the water were determined by dynamic light scattering (DLS), nanoparticle tracking analysis (NTA), and electrophoretic light scattering (ELS). Each measurement was performed at room temperature (*n* = 3–5). The morphology of nanoparticles was evaluated by transmission electron microscopy (TEM) using coper grids (300 mesh) with a carbon and formvar coating. 

#### 2.3.4. Drug Loading 

The effect of drug amount at constant polymer concentration: From 1 to 10 mg of DOD was dissolved in 2 mL ACN/THF = 1/1 (*v*/*v*) or THF. Subsequently, 20 mg of polymer in 2 mL of the same organic phase as DOD was added to the DOD solution to reach the ratio of 50 to 500 µg/mg of polymer. In this case, the polymer concertation was 5 mg/mL for the organic phase. The resulting solution was incubated for 1 h under stirring (420 rpm) at 37 °C and was then injected into 20 mL of deionized water (pH 6.2) or aqueous solutions that were prepared with adjusted pH values of 2.2, 3.2, 4.2, or 5.2 and 0.1 M HCl solution (850 rpm stirring, injection rate 2 mL/min). 

Effect of polymer concentration in organic phase at constant DOD/polymer ratio: Between 20 and 240 mg of PEG-*b*-PCL was dissolved in 2 mL of THF, and between 5 and 60 mg of DOD was also dissolved in 2 mL of THF. The solutions were mixed together to obtain a set of solutions with a DOD/polymer ratio equal to 250 μg/mg and a polymer concentration ranging from 5 to 60 mg/mL. The resulting solution was incubated for 1 h under stirring (420 rpm) at 37 °C and was then injected into 20 mL deionized water (pH 6.2) at a flow rate of 2 mL/min under stirring (850 rpm). 

To remove the organic solvent, the system was left open with stirring at 22 °C for 24 h. The resulting suspension of nanoparticles was purified from the non-encapsulated cytostatic drug and solvent traces by dialysis for 1–2 h. After dialysis, a series of 3 samples with a volume of 2 mL were freeze-dried and then dissolved in dichloromethane. The drug yield in the obtained solutions was determined spectrophotometrically at a wavelength of 260 nm with the use of a pre-built calibration plot (Figure S5, Supplementary Materials). Encapsulation efficacy (*EE*) and drug loading (*Q*) were calculated using Equations (1) and (2):

(1) $$EE=\frac{m_{loaded\;drug}}{m_{initial}}\times 100\%$$

(2) $$Q=\frac{m_{loaded\;drug}}{m_{polymer}}\times 100\%$$

#### 2.3.5. Release Study

GeBAflex Midi 50–800 µL dialysis tubes with an MWCO of 1000 and a sample volume of 0.7 mL were purchased from Scienova GmbH (Jena, Germany) and used for the release study. Before this, the permeability of the membrane towards DOD was tested. For this experiment, 20 μL of a DOD stock solution in THF (5 mg/mL) was added to 680 μL of deionized water in a dialysis tube, which was placed into a 50 mL plastic tube with a 12 mL solution of 0.01 M phosphate buffer containing 0.9% NaCl with a pH of 7.4 (PBS) (*n* = 3). The system was left for 24 h at 37 °C. After 1, 2, 4, 6, and 24 h, DOD transport through the membrane was 32, 52, 72, 92 and 100%, respectively.

To investigate the release of DOD, the PEG-*b*-PLA- and PEG-*b*-PCL-based nanoformulations were prepared as described in Section 2.3.4. The DOD loading for samples used in the release study was 136 µg/mg of PEG-*b*-PLA and 142 µg/mg of PEG-*b*-PCL. From the obtained purified suspension, a 700 µL aliquot was taken into an 800 µL dialysis tube and incubated, as described above for the free drug, at 120 rpm (*n* = 3 for each polymer). In each sample, the medium in the outer chamber was replaced at scheduled time intervals: 1, 2, 4, 6, 24, 48, 72, 144, and 192 h. The samples containing the released drug were analyzed by quantitative HPLC with mass-spectrometric detection (HPLC-MS). 

Chromatographic studies were performed using an HPLC-MS/MS LCMS-8030 system (upgraded to LCMS-8030Plus) with a tandem mass-selective detector (triple quadrupole, Shimadzu, Tokyo, Japan) equipped with a Kinetex^®^ C18 Phenomenex column (150 mm × 2.1 mm, 5 µm, 100 Å) (Torrance, CA, USA). The volume of the injected sample was 1 µL. Deionized water (18.2 MOhm/cm, purified using a D-301 deionizer, Aquilon, Russia) (eluent A) and acetonitrile (LC-MS grade, Panreac, Spain) (eluent B) were used as eluents. Both eluents were acidified with 0.1% formic acid (Formic acid LC-MS grade, Carlo Erba, Val de Reuil, France). The analysis was performed using a gradient mode at 40 °C. The flow rate of the mobile phase was 0.4 mL/min. The following elution program was used for analysis: 0–1 min, 5% B; 1.01–3.50 min, from 5 to 50% B; 3.51–7.00 min, 100% B; and 7.01–8.00 min, 5% B. 

Common mathematical dissolution models were applied for comparison of DOD release from the NPs under study. The linearization of experimental release profiles was carried out with the following dissolution models: zero-order, first-order, Higuchi, Hixson–Crowell, Korsmeyer–Peppas, Baker–Lonsdale, Hopfenberg, Weibull, Gompertz, and Peppas–Sahlin [30]. The DDSolver add-in for Microsoft Excel (freely available software which was developed by Zhang Yong and colleagues from China Pharmaceutical University [31]) was used for this purpose. The relevant correlation coefficients, kinetic constants, and model parameters were evaluated and analyzed to define the release mechanism.

#### 2.3.6. MTT Assay

Cells for the MTT assay were seeded in 96-well plates at an inoculation density of 18,000/cm^2^ for A2780 and 9000/cm^2^ for both SK-OV-3 and CHO-K1. After attachment, media were replaced with serum-free media and empty or DOD-loaded nanoparticles were added in the same range of concentrations via the double dilution method. As control samples, corresponding dilutions of PBS were used. After 72 h of exposure to samples, a 0.5 mg/mL MTT solution was added to each well and plates were incubated for 1.5 h. The medium was then removed, formazan crystals were dissolved in DMSO, and light absorbance at 570 nm was measured using a Tecan Infinite F50 (Tecan, Switzerland). 

#### 2.3.7. Study of Cell Cycles

The cells for the assay were seeded in 6-well plates at a seeding density of 9000/cm^2^ for both SK-OV-3 and CHO-K1. After cell attachment, the media were replaced with serum-free media. Subsequently, DOD-loaded or empty nanoparticles and a dioxadet solution at a concentration of 30 μg/mL were added. Sodium-phosphate buffer was used for control samples. After 24 h of exposure to the samples, the cells were separated and lysed for 30 min in a buffer containing 0.1% sodium citrate, 0.3% Triton-X100, 100 μg/mL RNase A, and 50 μg/mL propidium iodide. The fluorescence of the nuclear suspension was then measured using a CytoFlex flow cytometer (Beckman Coulter, Indianapolis, IN, USA).

#### 2.3.8. Statistical Analysis

The data are presented as mean value ± SDV (*n* = 4 for biological experiments; *n* = 3 for physicochemical experiments). Calculations of average particle size from TEM images were performed by analysis of 3 images for the same sample. The total number of analyzed particles ranged between 30 and 50. Data from biological experiments were analyzed by two-way ANOVA using Instat GraphPad Software (San Diego, CA, USA). *p* < 0.05 was considered statistically significant.

## 3. Results and Discussion

### 3.1. Synthesis of mPEG-b-PLA and mPEG-b-PCL

It is known that the molecular weight of biodegradable copolymers affects the physicochemical properties of the materials fabricated from them and determines key properties such as biodegradation rate [32,33] and drug release [34,35]. In this regard, the synthesis of copolymers with the specified characteristics is very important for their application in biomedicine. In order to study the effect of copolymer molecular weight on the characteristics and properties of nanoformulations, a number of copolymers with different molecular weights were synthesized and characterized.

The synthesis of copolymers with different molecular weights was carried out by ring-opening polymerization (ROP) in bulk (Figure S1, Supplementary Materials). The regulation of molecular weight was achieved by variation of polymerization time (60–90 min) and temperature (130–140 °C). The structures of polymers obtained were examined via ^1^H NMR spectroscopy. In the case of mPEG-*b*-PLA, signals corresponding to PLA and PEG were detected (Section 2.3.1, Figure S2, Supplementary Materials). In addition, PLA homopolymers were synthesized to compare the differences between mPEG-*b*-PLA and PLA in regard to the physicochemical properties of forming particles and the total loading of the drug. All copolymers were characterized using the SEC method to determine their molecular weight and dispersity (*Ɖ*) (Table 1).

In general, the introduction of mPEG into the polymerization mixture provided a decrease in mPEG-*b*-PLA copolymer molecular weight, which also occurred under various equal conditions. In particular, one can compare samples #4 and #10 (140 °C, 1.5 h) and samples #1 and #7 (130 °C, 1 h) and notice the growth in molecular weight from 10,000 to 26,200 for the first pair of polymer samples and from 36,400 to 178,000 for the second pair. Such a result may be associated with the increased number of initiation sites caused by elevated amounts of the co-initiator (mPEG-OH or H_2_O). In turn, this results in a greater number of macromolecules with smaller length. Another factor leading to the decrease in molecular weight in the presence of OH-containing compounds is the chain breakdown side reaction due to intermolecular or intramolecular nucleophilic attack of the growing polymer chain on ester moieties [36]. The same process occurs in presence of catalytic amounts of water in the reaction mixture (Table 1, samples #2 and #3). Similarly to our previous results, the addition of water and mPEG-OH decreased not only molecular weight, but also polymer yield [36]. 

In turn, an increase in polymerization temperature from 130 to 140 °C led to growth in the molecular weight of the resulting polymers. For instance, such a change in polymerization temperature was accompanied by an increase in molecular weight from 178,000 (sample #1) to 323,000 (samples #2) for PLA and 36,400 (sample #7) to 129,300 (sample #9) for mPEG-*b*-PLA. Thus, introduction of mPEG, as well as the addition of water, significantly reduces the molecular weight of resulting copolymers compared to just the PLA homopolymer. 

The greater hydrophobicity of PCL, which is related to longer degradation than that of PLA, has motivated us to synthetize mPEG-*b*-PCL with a lower molecular weight. The ratio of mPEG-OH to tin octoate in the synthesis of mPEG-*b*-PCL and addition of dodecanol as an initiator in the synthesis of PCL were varied for this purpose. The details and polymer characteristics are provided in Table 1. 

The structure and chemical composition of homopolymers and copolymers were confirmed by ^1^H NMR spectroscopy. Signals corresponding to both PCL and PEG were detected (Section 2.3.2, Figure S3, Supplementary Materials). According to the data obtained through SEC, the use of hydroxyl-containing compounds allowed for reductions in polymer chain length (Table 1, comparing sample #5 to samples #6, #11, and #12), which is in agreement with previously published data [36,37]. 

### 3.2. Preparation of Polymer Particles by Nanoprecipitation 

#### 3.2.1. Optimization of Preparation Techniques

The production of nanoparticles was performed via nanoprecipitation. The main driving force of the nanoprecipitation process is the fast supersaturation of the solution as a result of rapid mixing of the organic and aqueous phases [38,39]. When a solution of a hydrophobic polymer in an organic solvent (miscible with water) is mixed with a large amount of water, the limit regarding the thermodynamic solubility of the polymer is exceeded, resulting in spontaneous nucleation with further growth of nuclei leading to larger particles. A stable suspension of monodisperse polymer nanoparticles can be obtained in a narrow metastable region for a polymer/organic solvent/water mixture (Figure S4, Supplementary Materials). Outside of that region, there is either almost complete precipitation of the polymer in the form of large aggregates (at high concentrations of polymer and water and low concentrations of organic solvent), or complete dissolution of the polymer without the formation of a supersaturated solution (at low concentrations of polymer and with large volumes of organic solvent). In comparison to other methods used to obtain polymer particles with such polymers, such as oil-in-water or double emulsion techniques, nanoprecipitation at optimized conditions provides formation of the smallest nanoparticles (65–350 nm) with a narrow particle size distribution [40]. 

In order to obtain fine nanoparticles, factors such as the concentration of the initial components and the mixing rate, which affect the size of the resulting polymer particles, were investigated. Selection of the organic solvent was based on the solubility of the polymer and the drugs in it, the miscibility of the solvent with water, and the ease of its removal from the system after the formation of nanoparticles. Taking this into account, acetonitrile (ACN) and tetrahydrofuran (THF) were selected as organic phases to prepare nanoparticles from mPEG-*b*-PLA and mPEG-*b*-PCL. To exclude the possible effect of molecular weight, all optimization procedures were performed with (co)polymers of a close weight-average molecular weight (around 30,000). 

**Figure 3:**
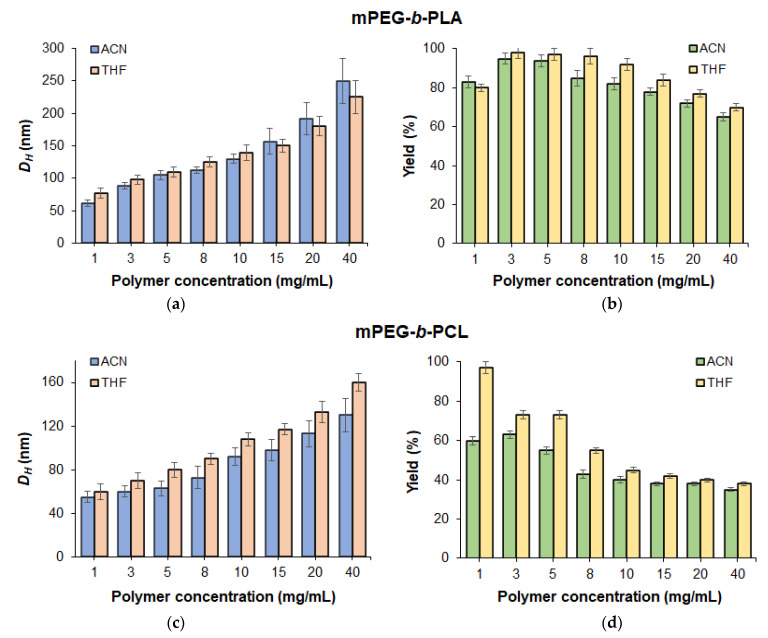
Dependence of the hydrodynamic diameter (**a**,**c**) and yield (**b**,**d**) of nanoparticles on the concentration of mPEG-*b*-PLA (**a**,**b**) and mPEG-*b*-PCL (**c**,**d**) (mixing rate: 850 rpm; aqueous phase: deionized water).

The dependence of nanoparticle size on the polymer concentration in ACN, ACN/THF mixtures, and THF was studied in concentrations ranging from 1 to 40 mg/mL (Figure 3). An increase in polymer concentration in organic phase accompanied by growth in the viscosity of the polymer solution provided an increase in the diameter of nanoparticles from 65 to 250 nm for mPEG-*b*-PLA (sample #7) and from 55 to 160 nm for mPEG-*b*-PCL (sample #11). At the same time, the yield of mPEG-*b*-PLA nanoparticles remained quite high even when obtained from very concentrated solutions (more than 60% in ACN and 70% for ACN/THF). In turn, the yield of mPEG-*b*-PCL nanoparticles significantly decreased when the polymer concentration in organic phase exceeded 5 mg/mL.

Fast mixing of the aqueous and organic phases is required in nanoprecipitation to achieve homogeneous supersaturation and produce monodisperse nanoparticles. In order to evaluate the effect of mixing rate, an mPEG-*b*-PLA solution with a concentration of 5 mg/mL and an aqueous phase to organic phase ratio equal to 5:1 was tested. The increase in mixing rate from 500 to 1000 rpm favored a decrease in the hydrodynamic diameter of nanoparticles from 149 to 107 nm (Figure S6, Supplementary Materials). Thus, the use of intensive mixing is favorable for obtaining nanoparticles of smaller size.

Using the optimized conditions for the preparation of nanoparticles, sets of nanoparticles from PLA, PCL, and their PEGylated copolymers were prepared and characterized by DLS, ELS, and NTA (Figure S7, Supplementary Materials). According to DLS, both mPEG-*b*-PLA and mPEG-*b*-PCL formed narrowly dispersed and monomodal nanoparticles (Figure S7) with negative ζ-potential. These results were in agreement with data obtained by NTA. However, being a more sensitive technique, NTA was able to detect some traces of nanoparticles at around 180–200 nm for mPEG-*b*-PCL (Figure S7). The results of the measurements of hydrodynamic diameters, polydispersity indexes, and ζ-potential for the different nanoparticles are summarized in Table 2.

The hydrodynamic diameters of polymer nanoparticles obtained by nanoprecipitation of PLA and mPEG-*b*-PLA were 135 and 115 nm, while PCL- and mPEG-*b*-PCL-based nanoparticles were around 150 and 100 nm in diameter, respectively. The introduction of PEG into a copolymer with PCL reduces particle size due to the formation of a compact core–shell structure. In the aqueous medium, a hydrophobic polymer is compacted inside the formed particles, but a hydrophilic mPEG block is located on the surface, thus increasing the stability of polymer carriers and preventing further particle growth. The decrease in the diameter of mPEG-*b*-PCL can be explained by the more hydrophobic nature of PCL, which leads to a denser packing of nanoparticles in water. The mean diameters of nanoparticles determined by both DLS and NTA were in agreement with each other. Moreover, all nanoparticles are characterized by low polydispersity index values (PDI < 0.1) and unimodal size distribution. Nanoparticles of PLA and mPEG-*b*-PLA are formed with high yields (76 and 95%, respectively). The yield for PCL and mPEG-*b*-PCL-based nanoparticles was lower (65 and 72%, respectively).

For comparison, Szczech and Szczepanowicz recently developed a method for preparing PLA and PCL nanoparticles of 80 and 76 nm, respectively, via spontaneous emulsification solvent evaporation involving the use of surfactants for the stabilization of nanoparticles [41]. The hydrodynamic diameter of nanoparticles obtained in this study for PLA and PCL was slightly greater, but nanoparticles did not contain a surface stabilizer as they did in the mentioned paper. In turn, Łukasiewicz et al. reported the preparation of PCL nanoparticles from both PCL and PEG-*b*-PCL via a nanoemulsion templating method [42]. The hydrodynamic diameter detected by DLS for both kinds of nanoparticles was ~250 nm (PDI < 0.2). At the same time, the nanoprecipitation technique optimized in this work allowed for the formation of nanoparticles two to three times smaller in size. 

#### 3.2.2. Stability of Nanoparticles 

In addition to their small size, all kinds of polymer nanoparticles possess a negative ζ-potential provided by the dissociation of surface-located carboxylic groups of PLA and PCL. Typical ζ-potential values for PLA and PCL nanoparticles were between −32 and −38 mV (Table 2). The introduction of mPEG into the copolymer structure led to a decrease in the absolute value of the ζ-potential due to a decrease in the number of surface-located carboxyl groups. In spite of this fact, the stability of nanoparticles bearing mPEG was not reduced. The stabilizing effect of PEG-corona is well known [43] and related to the high solubilization of hydrophilic polymers on the surface of the particles generating their repulsion. Furthermore, in vitro biological experiments revealed at least a two-fold reduction in the macrophage uptake of PEGylated PLA nanoparticles compared to PLA-based ones [44]. 

To evaluate the stability of PEGylated polymer particles, nanoparticles produced from copolymers with close molecular weights (*M_w_* = 36,400 for mPEG-*b*-PLA and *M_w_* = 29,200 for mPEG-*b*-PCL) were incubated in water and PBS (0.01 M phosphate buffer containing 0.15 mol/L NaCl). Monitoring of stability towards aggregation was carried out by DLS for dispersions incubated under conditions imitating storage (4 and 22 °C) and physiological temperature (37 °C). 

**Figure 4:**
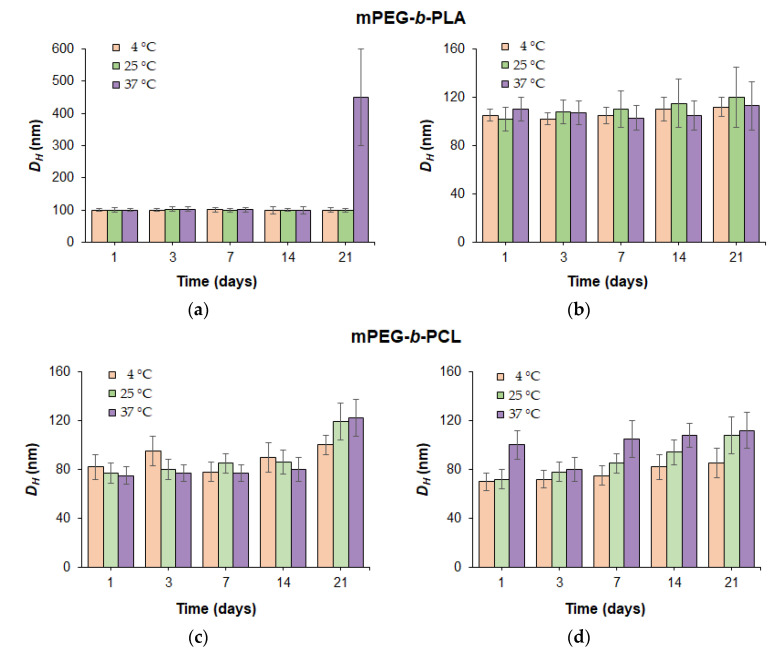
Dependence of the hydrodynamic diameters for mPEG-*b*-PLA (**a**,**b**) and mPEG-*b*-PCL (**c**,**d**) nanoparticles in deionized water (**a**,**c**) and 0.01 M PBS (pH 7.4) (**b**,**d**) on the incubation time at different temperatures.

At refrigeration and room storage temperatures (4 and 22 °C), the dispersions of the mPEG-*b*-PLA and mPEG-*b*-PCL nanoparticles in both water and PBS remained stable throughout the experiment (21 days) (Figure 4). Distribution of the hydrodynamic diameters of nanoparticles was rather narrow: PDI < 0.3. At 37 °C, both mPEG-*b*-PLA and mPEG-*b*-PCL dispersions were stable in PBS for 3 weeks. In turn, incubation of an mPEG-*b*-PCL dispersion at 37 °C in water demonstrated good hydrodynamic size stability (PDI < 0.3), while mPEG-*b*-PLA nanoparticles showed a tendency to aggregate on day 21 of incubation under similar conditions. 

It is well known that the more hydrophobic and semi-crystalline PCL is, the less degradable and therefore more stable it is [45,46]. Since both copolymers had close molecular weights, this result may be related to the lower degradation stability of mPEG-*b*-PLA due to its greater hydrophilicity and better water absorption compared to mPEG-*b*-PCL. Thus, the early stages of polymer degradation may be a driving force for the initiation of nanoparticle aggregation. 

For PEG-*b*-PLA, a similar trend in stability was recently observed by Maikawa et al., who used copolymers with similar molecular weight characteristics (*M_w_* = 32,770; *Ɖ* = 1.13) for the preparation of nanoparticles via nanoprecipitation from DMF to water [47]. The obtained PEG-*b*-PLA nanoparticles, with average *D_H_* equal to 57.6 nm, were incubated in deionized water at room temperature at 37 °C for 12 weeks and monitored by DLS. Throughout the experiment, no changes in the hydrodynamic diameters of the nanoparticles at room temperature were observed. In turn, incubation of PEG-*b*-PLA nanoparticles at 37 °C showed an initial increase in *D_H_* after 3 weeks. The slightly earlier aggregation in our case (after 2 weeks) compared to the reported data (after 3 weeks) can be attributed to the one and a half times larger diameter of our PEG-*b*-PLA particles with comparable polymer characteristics. 

### 3.3. Development of Dioxadet/mPEG-b-PLA and Dioxadet/mPEG-b-PCL Nanomedicines

The encapsulation of DOD was achieved through simultaneous nanoprecipitation of the copolymer and the drug during the formation of nanoparticles in water (Figure 1). This technique is widely used for the encapsulation of various poorly water-soluble and amphiphilic compounds (including anticancer compounds [48], antibiotics [49], dyes [47], etc.) into PLA and its copolymer-based nanoparticles [40]. To optimize the preparation of the nanoformulations, purification time after encapsulation was first optimized (Figure S8, Supplementary Materials). A time of 1 h was chosen as optimal. 

#### 3.3.1. Effect of Initial Drug Amount on the Encapsulation Efficacy and Characteristics of Nanomedicines

**Figure 5:**
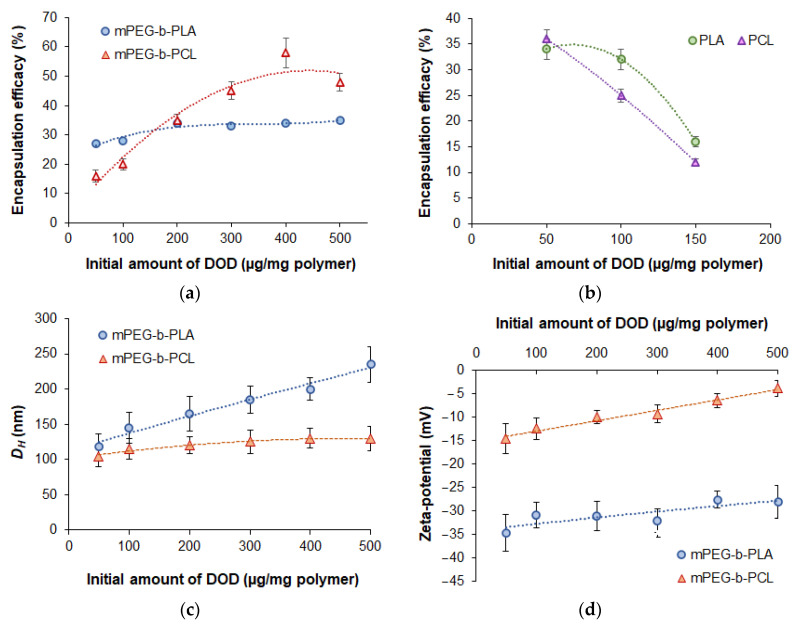
Dependence of the different characteristics of nanomedicines on the initial amount of DOD in the system: encapsulation efficacy for nanoformulations based on mPEG-*b*-PLA/mPEG-*b*-PCL (**a**) and PLA/PCL (**b**), in relation to hydrodynamic diameter (**c**), and in terms of ζ-potential (**d**). Characteristics of polymers and empty nanoparticles are provided in Table 2.

Figure 5 illustrates the dependence of encapsulation efficacy (*EE*) into nanoparticles on the type of polymer and the initial dioxadet amount at constant polymer concentration in organic phase (5 mg/mL). In all cases, encapsulation was carried out by nanoprecipitation from organic phase into deionized water. A slight increase in *EE* values for mPEG-*b*-PLA (from 27 to 35%) for mPEG-*b*-PCL (from 16 to 48%) was observed when the initial amount of DOD was increased from 50 to 500 μg/mg of polymer (Figure 5a). The higher drug loading into mPEG-*b*-PCL delivery systems is explained by the higher hydrophobicity of PCL in comparison to PLA. This facilitates the capture and retention of DOD in aqueous medium due to the greater affinity of drug hydrophobic moieties towards this polymer. 

For comparison, Chalermnon et al. recently reported the loading of 1,3,5-triazine derivatives with anticancer activity into calcium citrate nanoparticles [50]. Variation of the loading conditions allowed the authors to obtain a stable nanoformulation with a mean particle diameter (SEM) of 148 nm and 16% drug loading. Increasing drug loading to 18% was accompanied by aggregation of the prepared nanoformulation.

To evaluate the effect of PEGylation on encapsulation efficiency, the loading of DOD into PLA and PCL nanoparticles was investigated (Figure 5b). In this case, the maximal initial amount of DOD at which encapsulation appeared possible was 150 μg/mg of polymer. Further increases in the amount of DOD led to significant aggregation of loaded nanoparticles due to the increase in size accompanied by a lack of hydrophobic surface stabilization. The maximal drug loading determined for PLA and PCL was 84 and 126 μg/mg of polymer, which was considerably lower than the loading for PEGylated polymer systems. 

In addition, analysis of the hydrodynamic diameter and ζ-potential of the encapsulated nanoparticles was performed using DLS and ELS, respectively. As seen from Figure 5c, an increase in drug amount was accompanied by the growth of the hydrodynamic diameter of the nanoparticles. However, the increase in *D_H_* for DOD/mPEG-*b*-PLA was much more pronounced than for DOD/mPEG-*b*-PCL. The smaller size of mPEG-*b*-PCL-based DOD nanomedicines is probably a result of the denser packaging of the polymer with the drug due to the higher hydrophobicity of PCL compared to PLA. It also could result from the ability of PCL to form crystalline regions, which is not possible for D,L-PLA. Thus, PCL macromolecules are more compactly packed within the particles [29]. As an example, the distribution of *D_H_* for polymer particles loaded with DOD, as registered by NTA, is shown in Figure S9 (Supplementary Materials). 

In general, all systems obtained were characterized by *D_H_* < 250 nm and PDI < 0.3, which is appropriate for formulations intended for intravenous administration. According to previously published reports, nanoparticles smaller than 400 nm are applicable for this purpose, and the optimal size is about 10–200 nm [51]. In our case, except for the DOD/ mPEG-*b*-PLA nanoformulation obtained at an initial DOD concentration of 500 μg/mg of polymer, other samples had *D_H_* ≤ 200 nm (PDI ≤ 0.25) (Figure 5c). Thus, this satisfies the tightest requirements for the use of rigid nanoparticles as a delivery system [52,53]. 

As expected, all obtained nanomedicines had negative ζ-potential (Figure 5d). This negative charge showed the tendency to decrease together with increases in DOD loading. Despite this fact, the particles remain stable towards aggregation due to the effect of the PEG corona. 

The yield of loaded mPEG-*b*-PLA nanoparticles tended to decrease from 91 to 70% when the initial drug amount was increased from 50 to 500 µg/mg of polymer (Table S1, Supplementary Materials). In turn, for mPEG-*b*-PCL, the yield was almost independent of the initial drug amount and was in the range of 43–47%. The lower yield of mPEG-*b*-PCL-based delivery systems may be related to (1) the formation of a semicrystalline polymer matrix which does not provide distribution of DOD between macromolecules with the same efficacy as amorphous D,L-PLA and (2) the precipitation of the polymer in the aqueous medium during nanoparticle formation. The latter could be the result of both the higher hydrophobicity of the mPEG-*b*-PCL and the presence of a hydrophobic drug in the system, which in turn leads to a faster phase separation than in the case of mPEG-*b*-PLA. 

#### 3.3.2. Effect of Polymer Molecular Weight on Encapsulation Efficacy 

**Figure 6:**
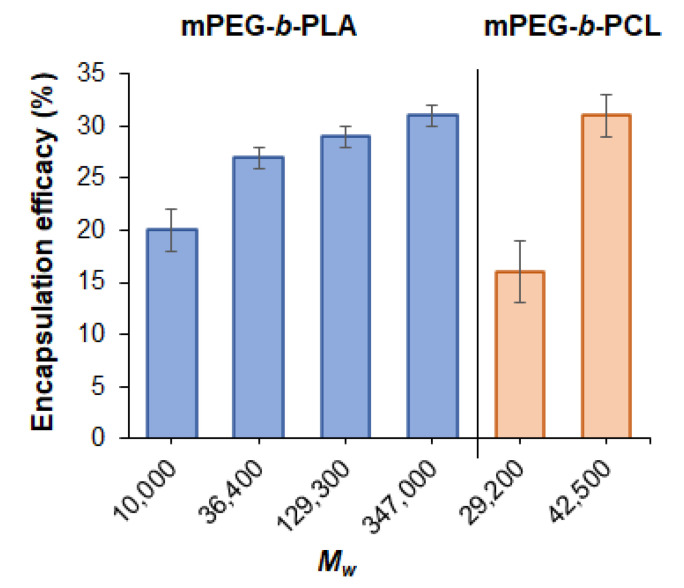
Dependence of the efficacy of dioxadet encapsulation into mPEG-*b*-PLA and mPEG-*b*-PCL nanoparticles on the molecular weight of the copolymer (the initial dioxadet amount was 50 µg/mg of polymer).

In order to study the effect of polymer molecular weight on encapsulation efficacy, a series of encapsulation experiments were performed under equal loading conditions: nanoprecipitation from ACN/THF for the DOD/mPEG-*b*-PLA system and from THF for DOD/mPEG-*b*-PCL in water at an initial DOD amount equal to 50 µg/mg of polymer and with a polymer concentration in organic phase of 5 mg/mL. The dependence of encapsulation efficacy on the molecular weight of polymers is presented in Figure 6. It can be seen that the elongation of the copolymer chains promotes the capture of a larger amount of the drug. This process was most pronounced, in the case of mPEG-*b*-PCL, when the molecular weight was increased from 29,200 to 42,500. However, further increases in molecular weight did not provide the formation of stable and narrowly dispersed nanoformulations due to uncontrolled precipitation of the copolymer. In the case of the DOD/mPEG-*b*-PLA system, the most evident increase in encapsulation efficacy was observed when the molecular weight of the copolymer was increased from 10,000 to 36,400. Further increases in molecular weight up to 347,000 provided only slight improvements in drug loading. 

#### 3.3.3. Effect of pH on Encapsulation Efficacy

**Figure 7:**
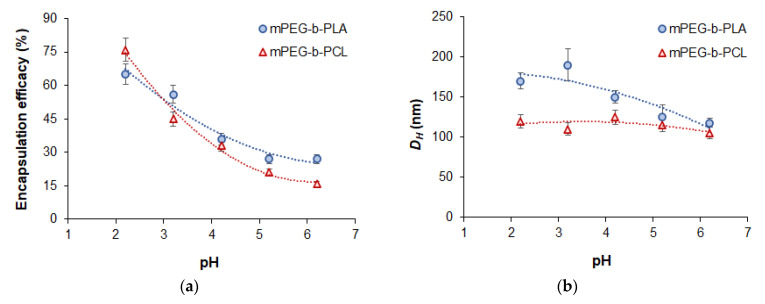
Effect of pH on the encapsulation efficacy (**a**) and hydrodynamic diameter (**b**) of the dioxadet delivery systems based on mPEG-*b*-PLA (*M_w_* = 36,400) and mPEG-*b*-PCL (*M_w_* = 29,200). The dioxadet/polymer ratio was 50 µg/mg of polymer.

Given that DOD contains several nitrogen atoms that can be protonated in an acidic medium, it would be reasonable to investigate the effect of the pH of the aqueous medium on DOD loading efficacy. For this purpose, the pH of the aqueous medium was varied from 6.2 (previously used water) to 2.2. Polymer concentration in organic phase in all cases was 5 mg/mL and the initial dioxadet amount was 50 µg/mg of polymer. As seen, the encapsulation efficacy for both copolymers increased when the pH became more acidic (Figure 7a). In turn, the hydrodynamic diameter of the mPEG-*b*-PCL-based nanoformulations remained at the same level, while the mPEG-*b*-PLA systems showed a slight increase in this parameter (Figure 7b).

Despite the high loading of DOD in solutions with a pH of 2.2, the nanomedicines obtained at this pH were characterized by very low stability towards aggregation during further manipulations, including purification. Such poor colloidal stability was the result of a loss of surface charge due to polymer protonation and partly due to compensation by the ionized drug. PEG detachment also may be a reason for that effect. In particular, the ζ-potential of nanoparticles loaded at a pH of 2.2 was −10.1 ± 2.4 mV for mPEG-*b*-PLA and 3.5 ± 1.9 mV for mPEG-*b*-PCL. At the same time, at a pH of 3.2, the nanoparticles were stable and had appropriate characteristics and high loading. For comparison, the ζ-potential of nanoparticles loaded at a pH of 3.2 was −20.8 ± 2.8 mV for mPEG-*b*-PLA and −3.0 ± 1.2 mV for mPEG-*b*-PCL. Therefore, the loading values obtained at this pH were selected for further comparison with loading at a pH of 6.2 (see Section 3.3.6). 

#### 3.3.4. Effect of Polymer Concentration in Organic Phase on Encapsulation Efficacy 

**Figure 8:**
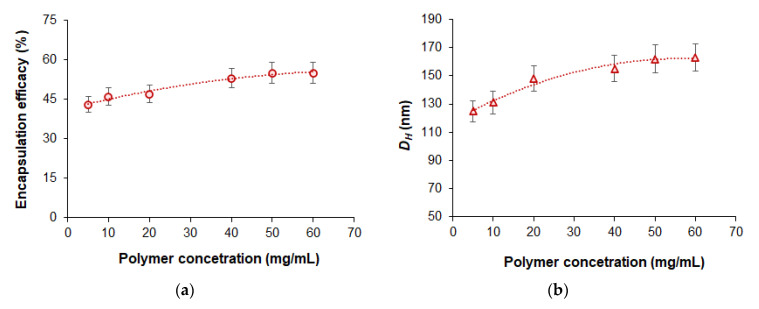
Effect of polymer concentration in organic phase on the encapsulation efficacy (**a**) and hydrodynamic diameter (**b**) of the dioxadet delivery systems based on mPEG-*b*-PCL (*M_w_* = 29,200). The dioxadet/polymer ratio was kept constant at 250 µg/mg of polymer.

In order to evaluate the effect of polymer concentration in organic phase on drug loading, we varied the concentration of mPEG-*b*-PCL from 5 to 60 mg/mL. Considering that the polymer mass increased significantly, we used a DOD/polymer ratio equal to 250 µg/mg of polymer in this experiment. It was found that increasing the concentration of the polymer solution used for nanoprecipitation from 5 to 60 mg/mL was accompanied by growth in encapsulation efficacy from 45 to 52% (Figure 8). At the same time, even at the highest polymer concentration in organic phase (60 mg/mL), the hydrodynamic diameter of the delivery system was 163 nm (PDI = 0.14).

#### 3.3.5. Comparison of Different Conditions for Dioxadet Loading

Table 3 summarizes the results regarding DOD encapsulation under different conditions. The initial amount of DOD relative to the polymer, pH, and the concentration of polymer in the organic phase was the key factor affecting drug loading. One can see that acidification of water to a pH of 3.2 was accompanied by an increase in encapsulation efficacy and drug loading. This effect was especially pronounced at a low initial amount of DOD (50 μg/mg) and was smoothed out when the initial amount was increased to 200 μg/mg. Increasing polymer concentration in the organic phase from 5 to 40 mg/mL at a fixed initial amount of DOD to polymer (250 μg/mg) resulted in a significant increase in encapsulation efficacy and drug loading. Almost the same loading can be achieved with a polymer concentration in the organic phase equal to 5 mg/mL, but with twice the amount of DOD to polymer (500 μg/mg). However, increasing polymer concentration allows for the preparation of concentrated dispersions that can be subsequently diluted to the desired level. The maximum DOD loading was 167 ± 9 μg/mg for mPEG-*b*-PLA (*M_w_* = 36,400) nanoparticles and 592 ± 22 μg/mg for mPEG-*b*-PCL (*M_w_* = 29,200) nanoparticles. Delivery systems with maximum DOD loading were characterized by a *D_H_* (PDI) of 222 nm (0.13) and 155 nm (0.12) for mPEG-*b*-PLA and mPEG-*b*-PCL, respectively. Thus, the more hydrophobic PCL-based PEGylated polymer provided three and a half times more loading at a smaller nanoparticle size than its PLA-based analogue. 

The importance of the hydrophobicity factor was also demonstrated for the encapsulation of DOD into nanoparticles formed by shorter chains of mPEG-*b*-PLA (*M_w_* = 10,000). For this, maximum DOD loading was twice as low (78 ± 6 μg/mg) as it was for its analogue with *M_w_* = 36,400. The PLA-block’s lack of hydrophobicity, required for nanoparticle compacting, contributed to the formation of a delivery system with a hydrodynamic diameter of 229 nm and a PDI of 0.16, even with relatively low maximum loading. 

#### 3.3.6. Morphology Study 

**Figure 9:**
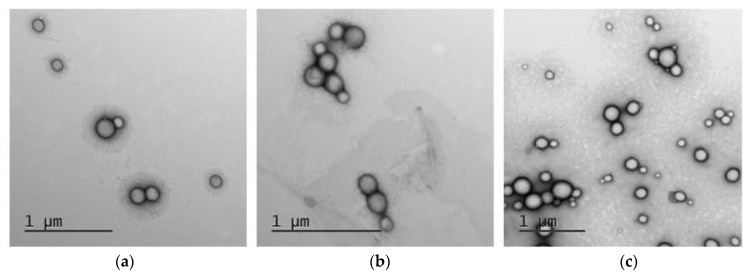
TEM images: empty mPEG-*b*-PLA nanoparticles (**a**); mPEG-*b*-PLA-based DOD nanoformulation (**b**); mPEG-*b*-PCL-based DOD nanoformulation (**c**).

TEM analysis was carried out with the use of uranyl acetate solution to contrast polymer nanoparticles. According to the images obtained, both empty and DOD-loaded nanoparticles were spherical (Figure 9). 

The diameter of empty mPEG-*b*-PLA and DOD/mPEG-*b*-PLA in a dry state was 113 ± 20 nm and 125 ± 35 nm, respectively. The hydrodynamic diameters for these nanoparticles were 115 nm (Table 2) and 118 nm (Table 3, the initial DOD amount was 50 μg/mg of polymer), respectively. In turn, DOD/mPEG-*b*-PCL nanoparticles in a dry state had a diameter of 104 ± 27 nm, which is also close to the DLS results: *D_H_* = 105 nm (Table 3, the initial DOD amount was 50 μg/mg of polymer). The similarity of the hydrodynamic diameter and the average particle diameter in the dry state indicates that the nanoparticles are dense solid nanospheres rather than micelles or other types of soft nanomaterials.

#### 3.3.7. Storage Stability of Developed Nanomedicines 

**Figure 10:**
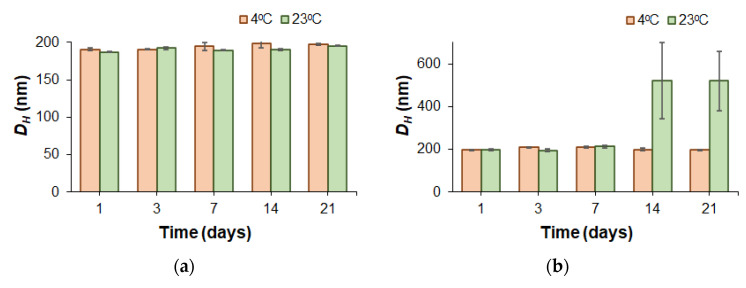

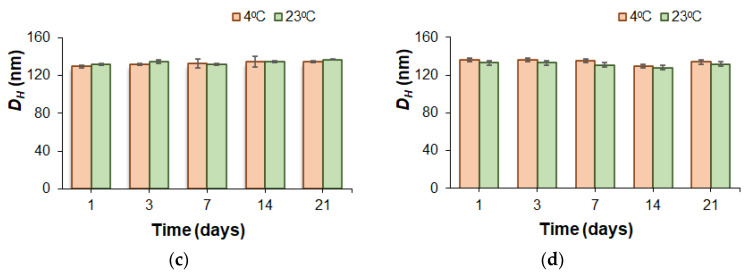
Storage stability of DOD/mPEG-*b*-PLA (**a**,**b**) and DOD/mPEG-*b*-PCL (**c**,**d**) nanomedicines in water (**a**,**c**) and 0.01 M PBS at a pH of 7.4 (**b**,**d**) at different temperatures. For the characteristics of the delivery system, see Table 3 (initial DOD amount, 400 μg/mg polymer).

The storage stability of DOD nanomedicines was evaluated in water and in 0.01 M PBS (pH 7.4) at 4 °C (refrigerator storage) and 23 °C (room temperature storage) over 3 weeks (Figure 10).

mPEG-*b*-PLA nanoparticles loaded with DOD were stable in both media at 4 °C and in water at 23 °C. Incubation of the DOD/mPEG-*b*-PLA nanoformulation at 23 °C in buffer was accompanied by significant aggregation as early as day 14 of storage. At the same time, the empty mPEG-*b*-PLA nanoparticles were stable at this temperature in buffer. Thus, after drug loading, only low-temperature storage guarantees the high stability of DOD/mPEG-*b*-PLA nanoformulations independent of the dispersion medium. In contrast, the DOD/mPEG-*b*-PCL system demonstrated high stability towards aggregation. In this case, hydrodynamic diameter and PDI remained constant in both water and buffer solution when incubated at 4 and 23 °C. The enhanced stability of mPEG-*b*-PCL nanoparticles over mPEG-*b*-PLA nanoparticles was also observed for empty nanoparticles (see Section 3.2.2).

### 3.4. In Vitro Release Study 

**Figure 11:**
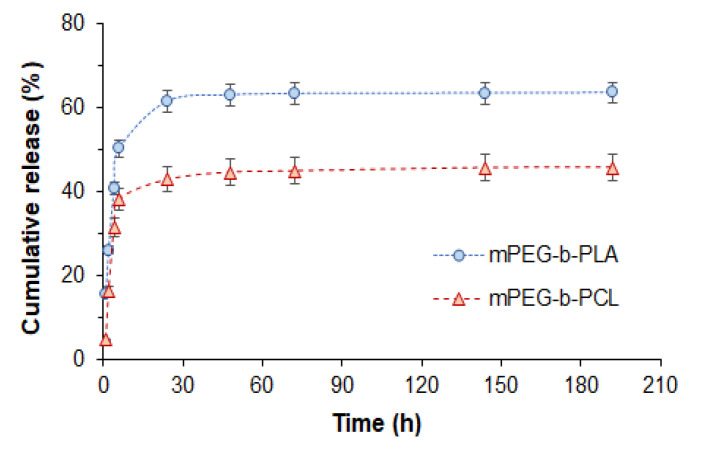
Profiles of DOD release from mPEG-b-PLA and mPEG-b-PCL (0.01 M PBS, pH 7.4).

The release of DOD from mPEG-*b*-PLA and mPEG-*b*-PCL was investigated by dialysis with the use of 0.01 M PBS (pH 7.4) as a medium. The cumulative release profiles shown in Figure 11 illustrate the faster release of DOD from mPEG-*b*-PLA nanoparticles. In particular, 64 and 46% release of DOD was achieved within 8 days by mPEG-*b*-PLA and mPEG-*b*-PCL nanoparticles, respectively. Both systems showed a burst release stage within 24 h (61 and 42% for mPEG-*b*-PLA and mPEG-*b*-PCL nanoparticles, respectively) that may be associated with the release of drug localized in the close-to-surface area and pores. After 24 h, both systems demonstrated a slow release, which is probably a degradation-dependent phase. 

Similar release profiles demonstrating a burst release within 24 h followed by slowed release were observed by Piazza et al. in a study of methotrexate/mPEG-*b*-PLA nanoformulations [54]. The authors detected a 16 to 40% release of methotrexate (an amphiphilic anticancer drug) from mPEG-*b*-PLA nanoparticles after 24 h depending on the copolymer’s composition and molecular weight. Mathematical processing of the release curves made it possible to conclude that non-Fickian diffusion was a main mechanism of those systems. 

Mishra et al. reported the release of 64 and >90% of docetaxel from mPEG-*b*-PLA nanoparticles in buffer medium with a pH of 7.4 after 24 h and 72 h, respectively [55]. A 100 nm formulation (PDI = 0.31) was also prepared by nanoprecipitation of the drug and copolymer (*M_w_* = 11,500, *Ɖ* = 1.15). The drug release results they obtained after 24 h were comparable to our findings with the same copolymer. The subsequent higher release of docetaxel compared to our results can be explained by the use of copolymers with different molecular weights and the length of the hydrophobic block (five times longer in our case).

As for release of 1,3,5-triazine derivatives, a 30% release from calcium citrate nanoparticles after 24 h and a 40% release after 48 h was observed in buffer medium (pH 7.4) by Chalermnon et al. [50]. 

To evaluate the possible mechanism for DOD release, the release profiles were analyzed through the application of common mathematical models (Table 4). One can observe that the full release profile could hardly fit to any of the standard models, the descriptive Weibull model being the only exception. At the same time, the first 10 h of release are quite well approximated by diffusion-based models such as the first-order and Higuchi models. These models reveal higher diffusion rate constants in the case of DOD release from mPEG-*b*-PLA compared to mPEG-*b*-PCL. The comparison of correlation coefficients shows better approximation of release from mPEG-*b*-PLA than from mPEG-*b*-PCL.

The good fitting with the Hopfenberg model allows us to conclude that release from the particles under study is associated with erosion and possesses biphasic release kinetics. The release exponent from Korsmeyer–Peppas model approximation shows that release of DOD within the first 10 h occurs according to anomalous non-Fickian transport. This means that both diffusion and polymer relaxation are important factors affecting the drug release process. This is in accordance with data approximation results using the Peppas–Sahlin model, in which *k*_1_ and *k*_2_ showed close values, revealing that diffusion and relaxation are both affecting drug release during the first 10 h. Application of this model for full release profiles shows that the whole process is mostly governed by diffusion (*k*_1_). 

The Weibull model is not associated with any mechanism of release but allows for evaluation of the time dependence parameter α and dissolution curve progression parameter β. The β values are below 1, which indicates the high initial slope of the exponential curve that is a characteristic of fast dissolving drugs. The time dependence parameter α shows very close values for both formulations.

Very good approximation with the Gompertz model argues in favor of the immediate release mechanism of well-soluble drugs. The β parameter from this model expresses the dissolution rate, which is quite similar for both of the formulations under study. This observation correlates with the fact that DOD is an amphiphilic substance, showing quite good solubility in water at low concentrations.

Summarizing all of the above, it can be concluded that the release of DOD from both formulations is governed by anomalous diffusion, which is affected by the relaxation of macromolecules. Such relaxation could be caused by the initial steps of degradation, as well as by the relaxation of PEG chains on the surface of the particles. The latter process is mostly expected during the early stage of drug release, while the former is anticipated at the late stage of drug dissolution. The overall process of DOD release from both formulations is characterized by biphasic kinetics with two different rates of anomalous diffusion (Figure S10, Supplementary Materials). 

### 3.5. In Vitro Biological Evaluation of Developed Nanomedicines

#### 3.5.1. Cytotoxicity Study 

**Figure 12:**
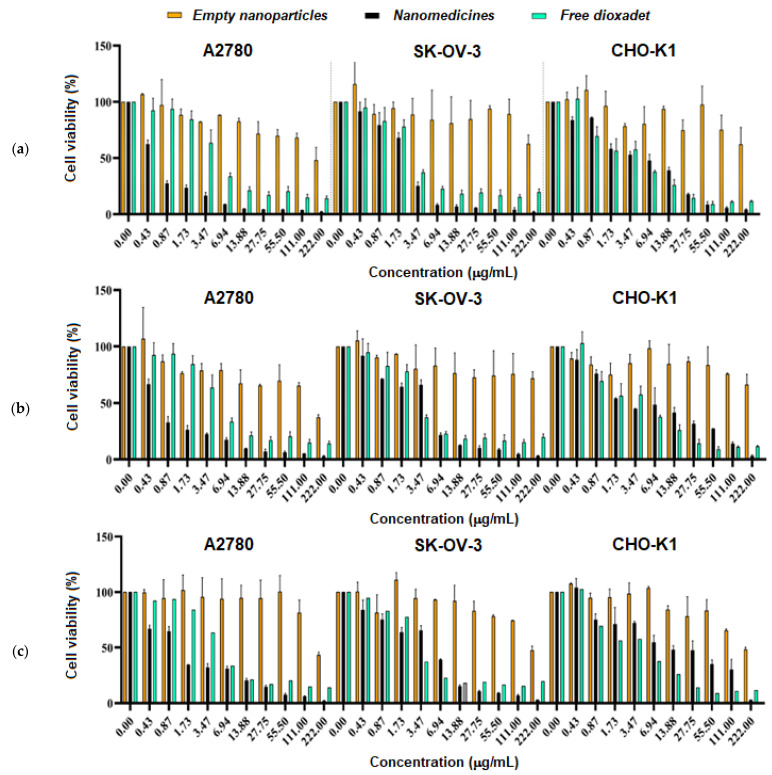
Cell viability of different cell lines in the presence of empty nanoparticles, nanomedicines, and free dioxadet. Empty nanoparticles and nanomedicines were obtained using the following polymers as a base: mPEG-*b*-PCL with *M_w_* = 29,200 (**a**), mPEG-b-PLA with *M_w_* = 36,400 (**b**), and mPEG-b-PLA with *M_w_* = 10,000 (**c**).

The viability of cells after 72 h of incubation with DOD nanomedicines or the free drug was evaluated by MTT assay (Figure 12). Three nanoformulations of DOD, based on mPEG-*b*-PLA with weight-average molecular weights of 10,000 and 36,400 and mPEG-*b*-PCL with *M_w_* = 29,200, were involved in the study of cytotoxicity. The Chinese hamster ovary cell line (CHO-K1A) was used as a control and two human ovarian adenocarcinoma cell lines, namely A2780 and SK-OV-3, were selected as the target cells. 

As expected, empty nanoparticles based on mPEG-*b*-PLA and mPEG-*b*-PCL did not demonstrate a cytotoxic effect up to a concentration of 111 μg/mL. In turn, free DOD and DOD nanoformulations demonstrated pronounced cytotoxic effects. No statistical difference was detected when comparing certain empty nanoparticles (mPEG-*b*-PLA with *M_w_* = 36,400, mPEG-*b*-PLA with *M_w_* = 10,000, or mPEG-*b*-PCL) in a range of different cell lines. In other cases, namely when comparing defined empty nanoparticles, nanoparticles loaded with DOD, and free DOD for different cell lines, the results were statistically different (*p* ≤ 0.001). 

The half-maximal inhibitory concentrations (IC_50_) of DOD for each system were calculated from a dose-dependent curve (Figure S11 of Supplementary Materials). According to the data obtained, free DOD was highly toxic to normal cells (CHO-K1) and cancer cells (SK-OV-3) (Table 5). In turn, all nanoformulations were as efficient as the free drug or even more effective against cancer cells. At the same, they were less toxic to normal cells. Comparing the different nanoformulations, one can deduce that the mPEG-*b*-PCL-based nanoformulation demonstrated the best efficiency (the lower IC_50_) towards cancer cells (*p* < 0.005). 

It is well known that the rate of nanoparticle uptake by cells depends on several factors, namely size, density, and surface charge [56,57,58]. In particular, positively charged and rigid nanoparticles are captured faster than neutral or negatively charged nanoparticles or soft nanostructures. In turn, smaller nanoparticles are better internalized than larger ones. In our case, all nanoparticles under study were negatively charged. Thus, this factor cannot be a reason for their different biological effect. At the same time, DOD-loaded nanoparticles based on mPEG-*b*-PCL (*D_H_* = 120 nm, PDI = 0.12) were smaller than the mPEG-*b*-PLA nanoparticles (*D_H_* = 222 nm, PDI = 0.13 for *M_w_* = 36,400 and *D_H_* = 228 nm, PDI = 0.16 for *M_w_* = 10,000) used in the cell experiments. Since smaller nanoparticles penetrate better into cells, this seems to be the reason for the higher cytotoxic effect of the DOD/mPEG-*b*-PCL nanoformulation.

#### 3.5.2. Study of Cell Cycles

**Figure 13:**
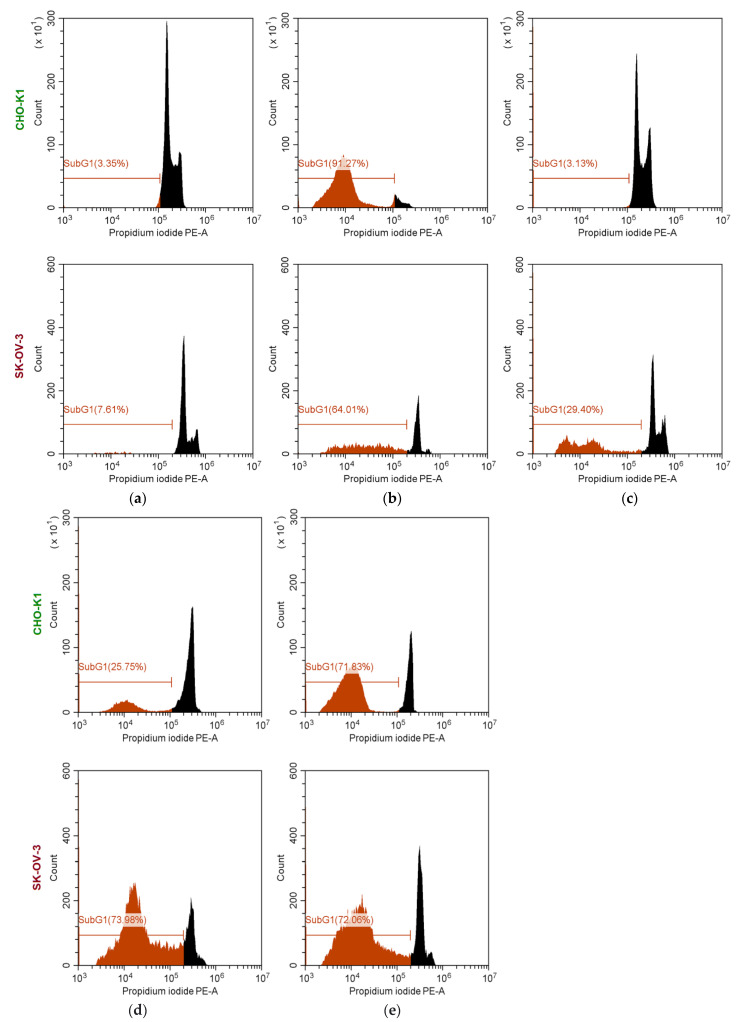
Study of the cell cycle (flow cytometry, 24 h) of untreated (**a**) and treated CHO-K1 (normal cells) and SK-OV-3 (cancer cells) (**b**–**e**). Cells were treated with free DOD (**b**), empty mPEG-*b*-PLA (*M_w_* = 36,400) nanoparticles (**c**), and DOD nanomedicines based on mPEG-*b*-PLA (*M_w_* = 36,400) (**d**) and mPEG-*b*-PCL (*M_w_* = 29,200) (**e**).

The study of cell cycle arrest is an additional way to assess the cytostatic effect of drugs. To observe noticeable effects after 24 h, the experiment was carried out with a concentration of DOD equal to 30 μg/mL. The use of concentrations exceeding IC_50_ is usually used to illustrate the phenotypic presence of an effect in the study of cell cycle arrest [59,60]. Using flow cytometry as a tool, the cell cycles of CHO-K1 (normal cells) and SK-OV-3 (cancer cells) were investigated for free DOD, empty PEG-*b*-PCL and PEG-b-PLA nanoparticles, and DOD nanomedicines based on the same copolymers. The results are shown in Figure 13. In the presented plots, it is possible to observe several peaks differing in intensity and color. Two peaks of different intensity, colored in black, correspond to (1) the G1 cell cycle region (which reflects the amount of DNA in the cells before division) (major peak) and (2) the G2 cell cycle region (which reflects the cells to divide) (minor peak). The area between peak 1 and 2 corresponds to the DNA replication phase. The red line and red peak indicate the SubG1 region corresponding to cells emitting less intensely than in the G1 region. The existence of the SubG1 region is explained by DNA fragmentation as a result of apoptosis. Therefore, the tendency for an increase in the number of cells in the SubG1 region and a decrease in the G1 and G2 peaks compared to the control sample indicates an increase in cell death. 

As seen from Figure 13a, the percentage of the SubG1 phase was around 3.4 and 7.6% for untreated CHO-K1 and SK-OV-3 cells, respectively. No toxic effect was revealed for normal cells and cancer cells treated with empty nanoparticles. As for untreated CHO-K1 cells, the area corresponding to the SubG1 phase was about 3% for cells treated with empty mPEG-*b*-PLA nanoparticles (Figure 13a,c). For SK-OV-3 cells, the SubG1 region was four times larger when the cells were incubated with nanoparticles; however, overall, the level of cell viability is acceptable. 

Incubation of cells with free DOD showed high toxicity for both normal cells and cancer cells. In particular, the SubG1 area was 91.3% for CHO-K1 and 64.0% for SK-OV-3 cells treated with free DOD. In turn, both DOD nanomedicines were toxic to cancer cells (the SubG1 area was around 72–74%) and their cytotoxicity exceeded the cytotoxicity of free DOD by 8–10%. At the same time, both DOD nanomedicines were less toxic to normal cells compared to free DOD: the SubG1 area was 25.8 and 71.8% for DOD/mPEG-*b*-PLA and DOD/mPEG-*b*-PCL, respectively. The higher cytotoxicity of the mPEG-*b*-PCL-based DOD nanoformulation can be attributed to probable faster uptake by cells due to smaller particle size.

The lower cytotoxicity of free DOD towards normal cells compared to cancer cells after 48 h was recently reported by Mikolaichuk et al. [15]. In particular, DOD demonstrated a slight decrease in the survival of human embryonic kidney cells (HEK 293), while pronounced dose-dependent cytotoxicity was observed for several cancer cells (liver (SK-HEP-1), as well as lung (A-549), ovarian (PA-1), and pancreatic (PANC-1) adenocarcinomas and glioblastoma (T98G)). 

## 4. Conclusions

This study presents results on the development of dioxadet-bearing nanomedicines based on mPEG-*b*-PLA and mPEG-*b*-PCL. To obtain nanomedicines, copolymers of different molecular weight were synthesized and characterized. It was established that PEGylation promotes the formation of smaller nanoparticles during nanoprecipitation and provides stabilization. An increase in the molecular weight of copolymers contributes to an increase in the hydrodynamic diameter of the nanoparticles. The intense stirring during nanoprecipitation allows for the formation of smaller nanoparticles. Loading of DOD, and increasing its initial amount from 50 to 500 μg/mg of polymer, leads to an increase in hydrodynamic diameter from 90 nm to 235 nm for mPEG-*b*-PLA and from 76 to 130 nm for mPEG-*b*-PCL nanoformulations. Encapsulation efficacy increases with elongation of the copolymer chain. Acidification of water to a pH of 3.2 was accompanied by an increase in encapsulation efficacy and drug loading at a low initial amount of DOD, which smoothed out when the initial amount of DOD was increased. At the same time, an increase in the concentration of polymer in the organic medium allows for the preparation of concentrated dispersions of nanomedicines with high DOD loading. 

According to TEM, DLS, and NTA analysis, formed nanoparticles are solid dense nanospheres. Empty and loaded mPEG-*b*-PCL nanoparticles demonstrated excellent stability over 3 weeks in water and buffer solution under conditions imitating refrigeration and room storage. Empty mPEG-*b*-PLA nanoparticles were also stable under the same conditions, while the DOD/mPEG-*b*-PLA nanoformulation was stable under low temperature (4 °C) in both water and buffer and at room temperature in water. The release of DOD demonstrates a characteristic burst for 24 h and then slows down significantly. The main driving force for release is anomalous diffusion, which is affected by the relaxation of macromolecules. 

Biological testing revealed that the empty nanoparticles were non-toxic to both normal cells (CHO-K1) and ovarian cancer (SK-OV-3 and A2780) cells. In general, the DOD nanomedicines demonstrated high in vitro efficacy, which was comparable to the free drug. Importantly, the developed nanomedicines were less toxic to normal cells than to cancer cells. Among the developed systems, the DOD/mPEG-*b*-PCL nanoformulation demonstrated the highest cytostatic effect. This may be attributed to better cell penetration due to the smaller size of the mPEG-*b*-PCL delivery systems compared to mPEG-*b*-PLA. Summarizing the results of the biological evaluations performed with different methods, we can conclude that encapsulated DOD retains its high cytotoxic effect on cancer cells but demonstrates reduced cytotoxicity to normal cells. This result is of great importance for the further potential application of anticancer drugs in vivo.

## Figures and Tables

**Table 1:** Effect of polymerization temperature and time of reaction on the yield and molecular weight of synthesized (co)polymers. Conditions: PLA and mPEG-*b*-PLA synthesis, [D,L-LD]:[Sn(Oct)_2_] = 1000, [D,L-LD]:[mPEG-OH] = 1500; PCL synthesis, [ε-CL]:[Sn(Oct)_2_] = 1000; mPEG-*b*-PCL synthesis, [ε-CL]:[Sn(Oct)_2_] = 1000, [ε-CL]:[mPEG-OH] = 190 or 380; D,L-LD was dried before polymerization overnight under vacuum at 30 °C.

Sample	Temperature (°C)	Time (h)	*M_w_*	*Ɖ*	Yield (%)
**PLA**					
#1	130	1	178,000	2.31	77
#2	140	1	323,000	1.33	88
#3 ^a^	140	1	7000	1.14	76
**#4 ^a^**	**140**	**1.5**	**26,200**	**1.13**	**78**
**PCL**					
#5	140	24	69,000	1.50	95
**#6 ^b^**	**140**	**24**	**30,000**	**1.67**	**82**
**mPEG-*b*-PLA**					
**#7**	**130**	**1**	**36,400**	**1.20**	**71**
#8	130	1.5	347,200	1.90	73
#9	140	1	129,300	1.95	66
#10 ^a^	140	1.5	10,000	1.67	70
**mPEG-*b*-PCL**					
**#11 ^c^**	**140**	**4**	**29,200**	**1.51**	**88**
#12 ^d^	140	24	42,500	1.54	87

^a^ Non-dried monomer containing water, [D,L-LD]:[H_2_O] = 310; ^b^ Dodecanol-1 was used as a co-initiator, [Dodecanol-1]:[Sn(Oct)_2_] = 1.9, [ε-CL]:[Dodecanol-1] = 510; ^c^ [mPEG-OH]:[Sn(Oct)_2_] = 5.2; ^d^ [mPEG-OH]:[Sn(Oct)_2_] = 2.6.

**Table 2:** Characteristics of the polymer nanoparticles obtained under optimized conditions.

Polymer	*M_w_*	DLS		NTA	ζ-Potential (mV)	Yield (%)
		*D_H_* (nm)	PDI	*D_H_* (nm)		
PLA	26,200	135 ± 43	0.09	143 ± 38	−37.5 ± 6.8	76
mPEG-*b*-PLA	36,400	115 ± 39	0.09	120 ± 27	−32.8 ± 5.0	95
PCL	30,000	148 ± 42	0.08	153 ± 49	−32.3 ± 6.6	65
mPEG-*b*-PCL	29,200	100 ± 28	0.08	104 ± 25	−22.6 ± 5.9	72

**Table 3:** Comparison of the results for DOD encapsulation depending on the loading conditions.

Encapsulation Conditions			DOD/mPEG-*b*-PLA			DOD/mPEG-*b*-PCL		
Aqueous Phase	C_polymer_ in Organic Phase (mg/mL)	Initial DOD/ Polymer (μg/mg)	*EE* (%)	Yield (%)	Drug Loading (μg/mg Polymer)	*EE* (%)	Yield (%)	Drug Loading (μg/mg Polymer)
Water, pH 6.2	5	50	23	74	16	16	44	17
Water, pH 3.2	5	50	68	70	49	45	33	70
Water, pH 6.2	5	200	34	72	74	34	46	140
Water, pH 3.2	5	200	−	−	−	26	33	151
Water, pH 6.2	5	250 500	33 30	79 70	91 167	44 48	45 43	243 592
Water, pH 6.2	40	250	37	56	152	53	30	545

**Table 4:** Correlation coefficients and model parameters obtained for DOD release profiles of mPEG-*b*-PLA and mPEG-*b*-PCL.

Model	Time (h)	mPEG-*b*-PLA		mPEG-*b*-PCL	
		R^2^	Parameters	R^2^	Parameters
Zero-order *F = k*_0_ *× t*	200	0.5907	*k*_0_ = 0.332	0.5624	*k*_0_ = 0.238
	10	0.9756	*k*_0_*=* 9.342	0.9837	*k*_0_ = 6.871
First-order *F =* 100 *×* [1 − *Exp*(−*k*_1_ *× t*)]	200	0.8603	*k*_1_*=* 0.029	0.6303	*k*_1_ = 0.004
	10	0.9931	*k*_1_ = 0.129	0.9902	*k*_1_ = 0.085
Higuchi *F = k_H_ × t*^0.5^	200	0.7419	*k_H_* = 5.450	0.7061	*k_H_ =* 3.898
	10	0.9942	*k_H_ =* 19.786	0.9556	*k_H_* = 14.143
Korsmeyer–Peppas *F* = *k_KP_ × t^n^*	24	0.9663	*k_KP_* = 23.240 *n* = 0.325	0.9126	*k_KP_* = 15.167 *n* = 0.358
	10	0.9991	*k_KP_* = 16.669 *n* = 0.624	0.9869	*k_KP_* = 8.279 *n* = 0.881
Hixson–Crowell *F* = 100 *×* [1 − (1 − *k_HC_ × t*)^3^]	200	0.7532	*k_HC_* = 4.2 × 10^−3^	0.6228	*k_HC_* = 1.2 × 10^−3^
	10	0.9886	*k_HC_* = 3.9 × 10^−2^	0.9888	*k_HC_* = 2.6 × 10^−2^
Hopfenberg *F* = 100 *×* [1 − (1 − *k_HB_ × t*)*^n^*]	200	0.8430	*k_HB_* = 6.2 × 10^−6^ *n* = 4593	0.6466	*k_HB_ =* 6.2 × 10^−6^ *n* = 693
	10	0.9931	*k_HB_* = 8.8 × 10^−5^	0.9902	*k_HB_* = 5.1 × 10^−4^
Baker–Lonsdale 3/2 *×* [1 − (1 − *F*/100)^2/3^] − *F*/100 = *k_BL_ × t*	200	0.8143	*k_BL_* = 1.1 × 10^−3^	0.7705	*k_BL_* = 3.8 × 10^−4^
	10	0.9892	*k_BL_* = 8.0 × 10^−3^	0.9487	*k_BL_* = 3.8 × 10^−3^
Peppas–Sahlin *F = k*_1_ *× t^m^ + k*_2_ *× t*^(2 *× m*)^	200	0.9738	*k*_1_*=* 28.476 *k*_2_ = 2.997 *m* = 0.327	0.9446	*k*_1_ = 18.784 *k*_2_ = 1.809 *m* = 0.344
	10	0.9999	*k*_1_*=* 60.457 *k*_2_ = 76.047 *m* = 0.148	0.9992	*k*_1_ = 576.440 *k*_2_ = 580.813 *m* = 0.030
Weibull *F* = 100 *×* {1 − *Exp*[−((*t* − *Ti*)*^β^*^)/*α*^]}	200	0.9734	*α* = 2.228 *β* = 0.172 *Ti* = 0.996	0.9933	*α* = 2.463 *β* = 0.083 *Ti* = 2.000
	10	0.9999	*α =* 4.946 *β =* 0.709 *Ti =* 0.216	0.9988	*α =* 5.794 *β =* 0.641 *Ti =* 0.873
Gompertz *F =* 100 *× Exp*{−*α × Exp*[−*β × log*(*t*)]}	200	0.9625	*α =* 1.392 *β =* 0.575	0.9245	*α =* 1.741 *β =* 0.398
	10	0.9990	*α* = 1.918 *β* = 1.286	0.9979	*α* = 2.847 *β* = 1.433

**Table 5:** IC_50_ values determined for free dioxadet and its nanomedicines based on mPEG-*b*-PLA and mPEG-*b*-PCL for three different cell lines.

Cell Line	IC_50_ (μg/mL)			
	Free DOD	DOD Nanomedicines Based on Copolymer:		
		mPEG-*b*-PCL (*M_w_* = 29,200)	mPEG-*b*-PLA (*M_w_* = 36,400)	mPEG-*b*-PLA (*M_w_* = 10,000)
CHO-K1	2.60	5.19	2.06	9.36
A2780	4.14	0.47	0.52	1.16
SK-OV-3	2.59	2.41	4.26	4.98

## Data Availability

Data available within the article or its Supplementary Materials.

## Supplementary Materials

The following supporting information can be downloaded at: https://www.mdpi.com/article/10.3390/pharmaceutics14112506/s1. Figure S1. Synthesis of PEG-*b*-PLA (a) and PEG-*b*-PCL (b) via ring-opening polymerization (ROP) of corresponding monomers. Figure S2. ^1^H NMR spectra for PLA (1) and PEG-*b*-PLA (2). Figure S3. ^1^H NMR spectra for PCL (a) and PEG-*b*-PCL (b). Figure S4. Three-component phase diagram of polymer/organic solvent/water with selected optimal wt% of components to prepare nanoparticles up to 100 nm in diameter. Figure S5. Calibration plot of absorbance on the different concentrations of dioxadet solutions (in dichloromethane). Figure S6. Dependence of the hydrodynamic diameter of mPEG-*b*-PLA nanoparticles on the mixing rate of the organic (ACN/THF) and aqueous (deionized water) phases. Figure S7. Distribution of mPEG-*b*-PLA (a,c) and mPEG-*b*-PCL (b,d) nanoparticles by hydrodynamic diameter and analyzed by DLS (a,b), and NTA (c,d). Figure S8. Dependence of the purification time of the prepared nanoformulations on encapsulation efficacy. Figure S9. Distribution of the hydrodynamic diameters of the nanomedicines (NTA; samples prepared at the initial DOD amount equal to 50 µg/mg polymer: (a) DOD/mPEG-*b*-PLA; (b) DOD/ mPEG-*b*-PCL; the red line represents the average of three measurements). Figure S10. Linearization of early and late DOD release from mPEG-*b*-PLA (a) and mPEG-*b*-PCL (b) as predicted with the Higuchi model. Quite clear linearizations show two stages with two different diffusion coefficients. Figure S11. Dependence of cell viability on the concentration of nanomedicines, empty polymer nanoparticles, and free dioxadet for different cell lines: (a) a series for mPEG-*b*-PCL; (b) a series for mPEG-*b*-PLA (*M_w_* 36,400), and (c) a series for mPEG-*b*-PLA (*M_w_* 10,000). Table S1. Dependence of the yield of nanoparticles based on mPEG-*b*-PLA (sample #7, Table 1) and mPEG-*b*-PCL (sample #11, Table 1) (nanoprecipitation from organic phase to water).
