# Supplementary material for: Nanomedicines Bearing an Alkylating Cytostatic Drug from the Group of 1,3,5-Triazine Derivatives: Development and Characterization
Source: Pharmaceutics. 2022 Nov 18;14(11):2506. doi: 10.3390/pharmaceutics14112506 (PMC9694467; doi:10.3390/pharmaceutics14112506)
Supplement: Supplementary file 1 [file pharmaceutics-14-02506-s001.zip › pharmaceutics-1997730-supplementary.pdf]

pharmaceutics

MDPI

Supplementary Materials

Nanomedicines Bearing an Alkylating Cytostatic Drug from the Group of 1,3,5-Triazine Derivatives: Development and Characterization

Ekaterina Sinitsyna [1,2], Irina Bagaeva [2], Erik Gandalipov [3], Evgenia Fedotova [2], Viktor Korzhikov-Vlakh [1,2], Tatiana Tennikova [2], Evgenia Korzhikova-Vlakh [1]

[1] Institute of Macromolecular Compounds, Russian Academy of Sciences, Bolshoy pr. 31, St. Petersburg 199004, Russia;
[2] Institute of Chemistry, Saint-Petersburg State University, Universitetsky pr. 26, St. Petersburg 198504, Russia;
[3] International Institute of Solution Chemistry and Advanced Materials Technologies, ITMO University, Lomonosov street 9, St. Petersburg 191002, Russia;

* Correspondence: vlakh@hq.macro.ru (E.K.-V.)

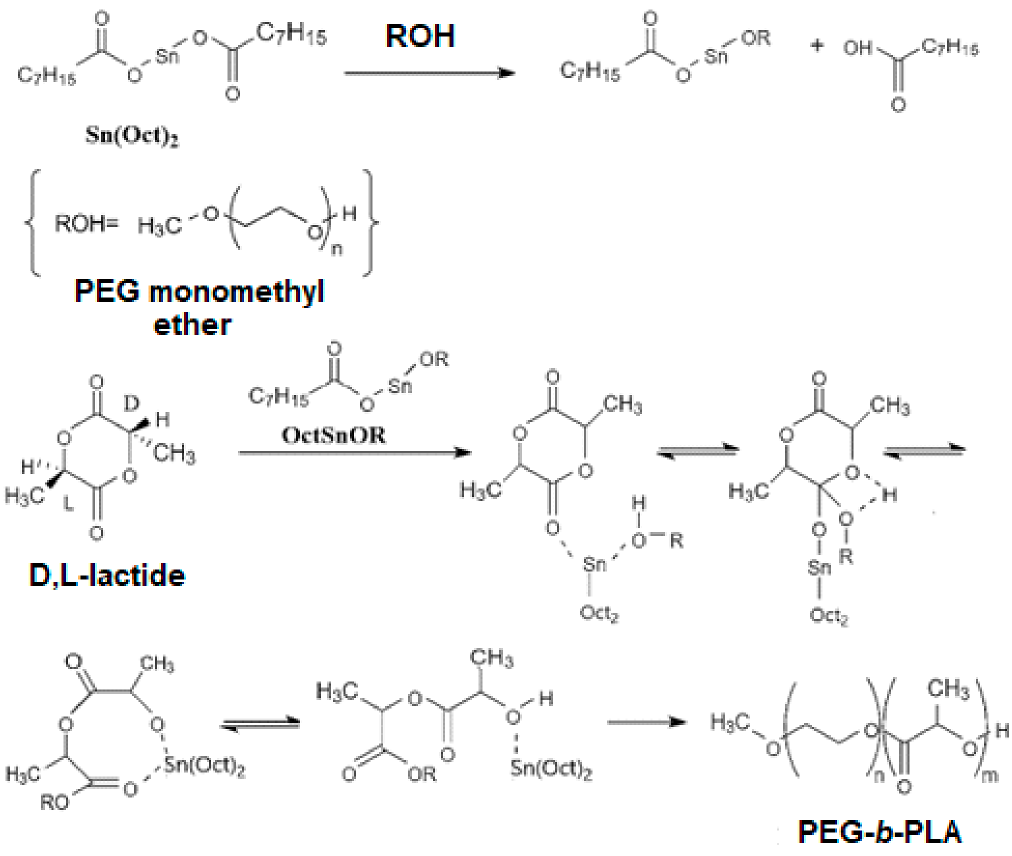

(a)

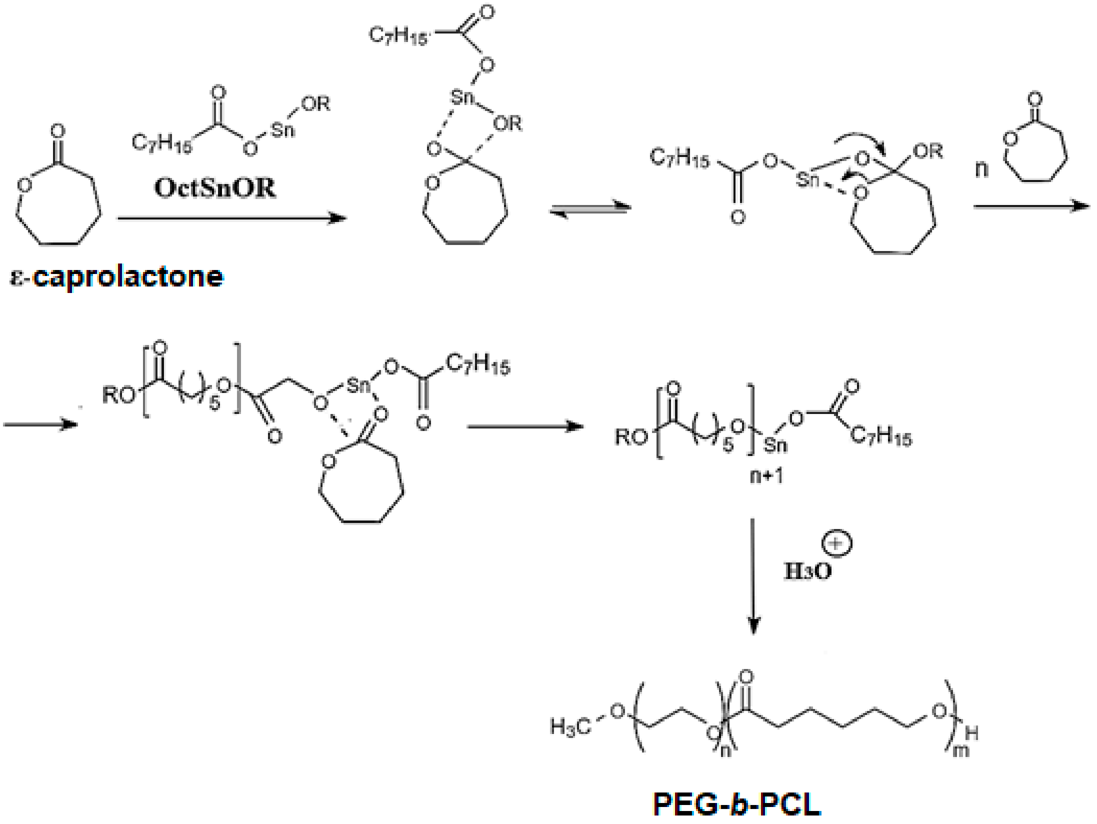

(b)

Figure S1. Synthesis of PEG-*b*-PLA (**a**) and PEG-*b*-PCL (**b**) *via* ring opening polymerization (ROP) of corresponding monomers.

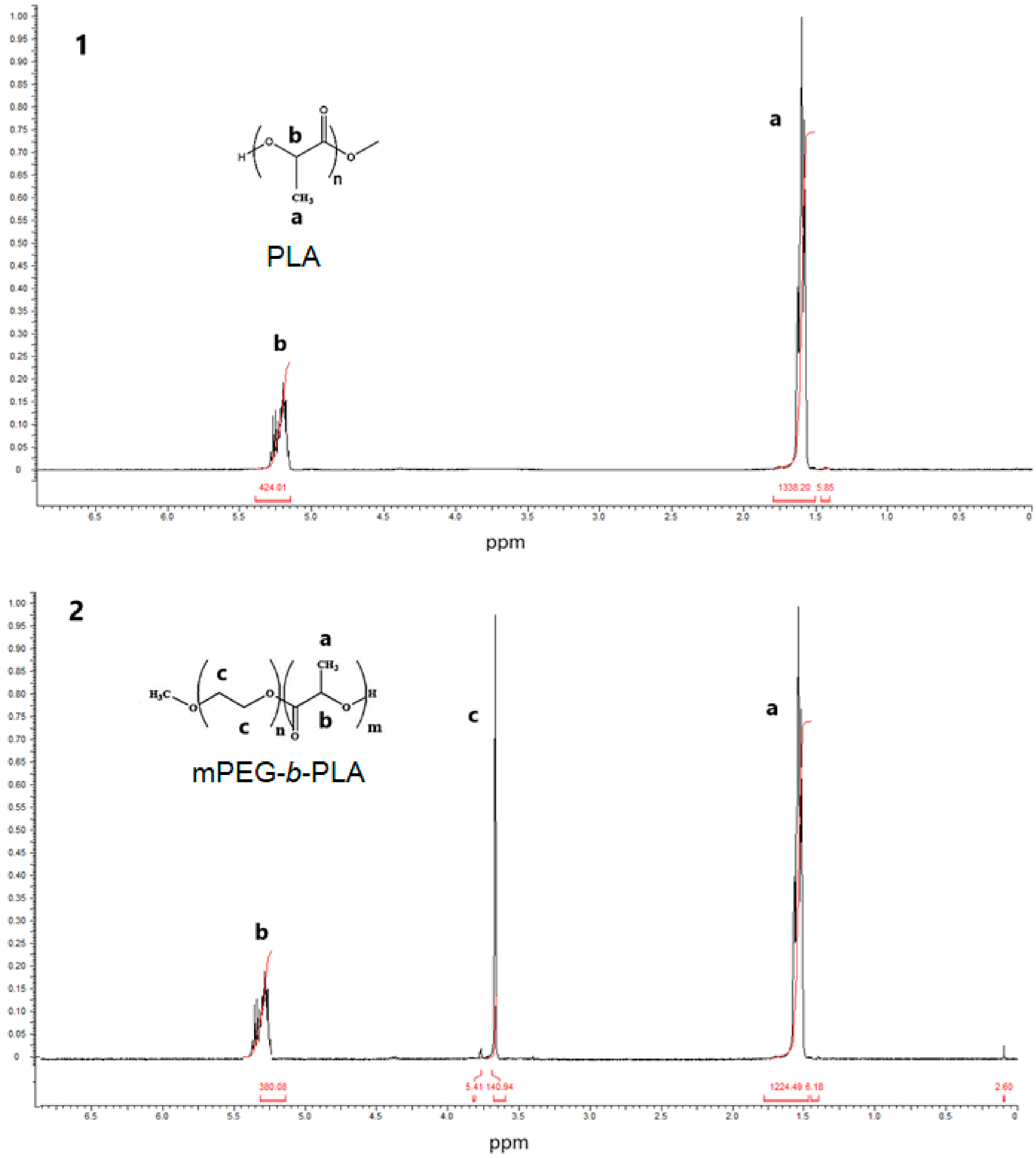

Figure S2. ^{1}H NMR spectra for PLA **(1)** and PEG-*b*-PLA **(2)**.

(a)

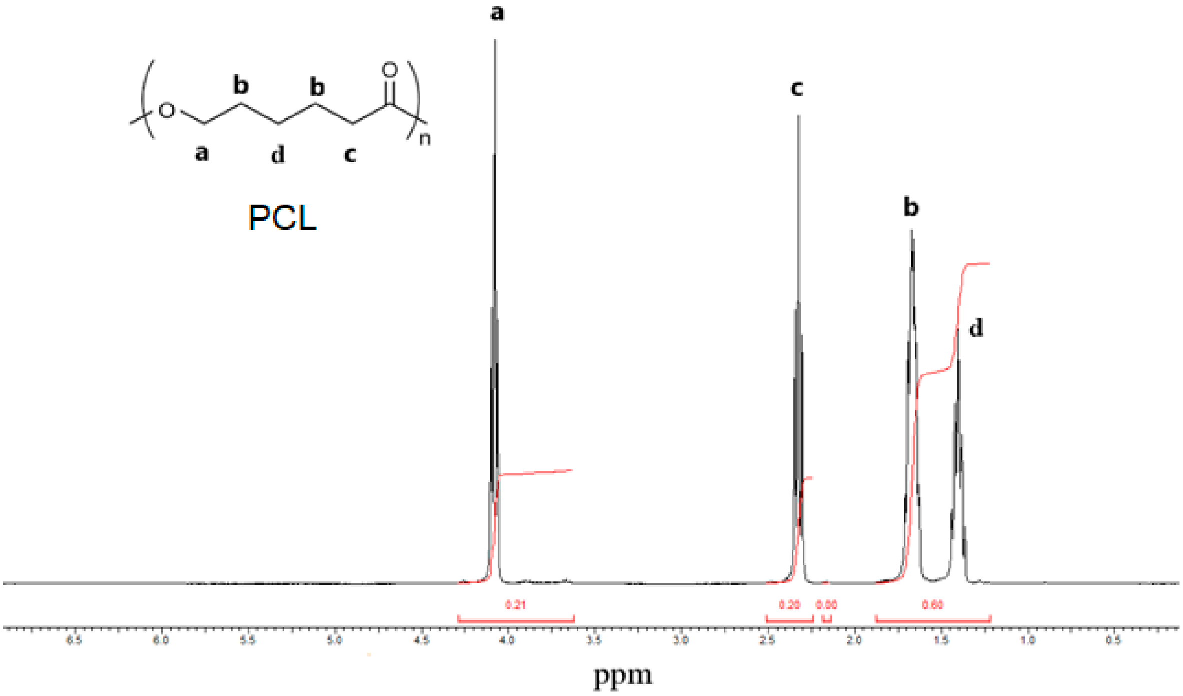

(b)

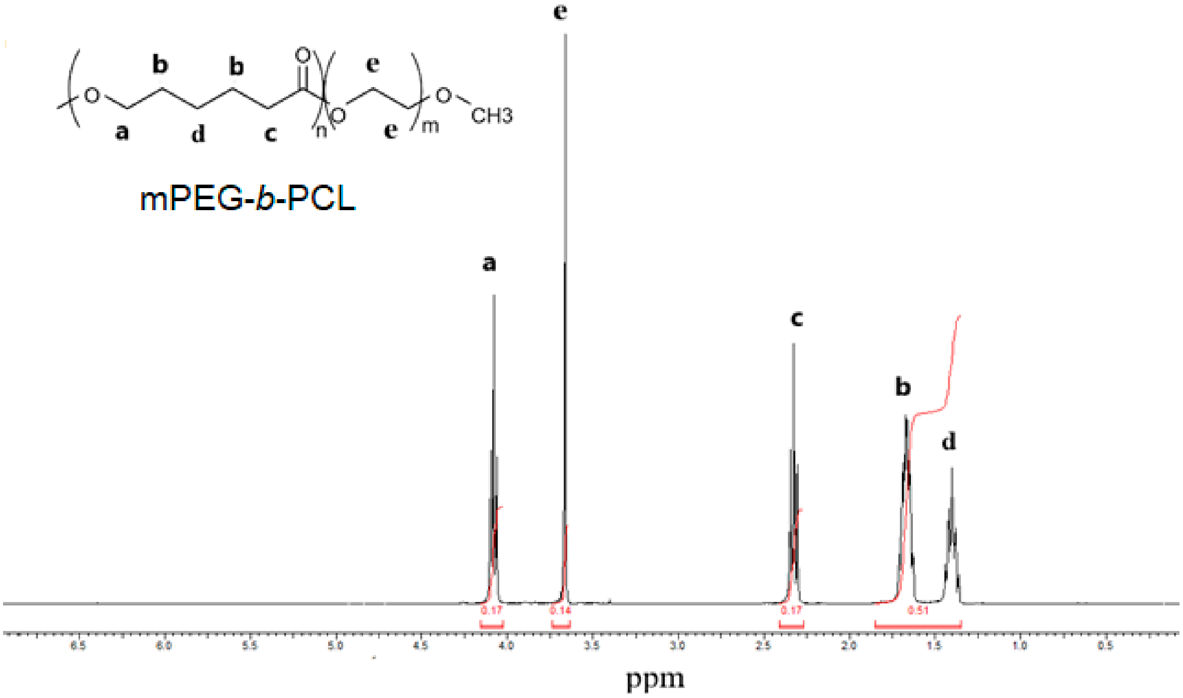

Figure S3. ^{1}H NMR spectra for PCL **(a)** and PEG-*b*-PCL **(b)**.

Using the three-component polymer/solvent/water phase diagram shown in **Figure S4**, the optimal composition of the initial components falling in the metastable region was as follows (all in wt%): polymer/acetonitrile/water = 0.08/16.7/83.2.

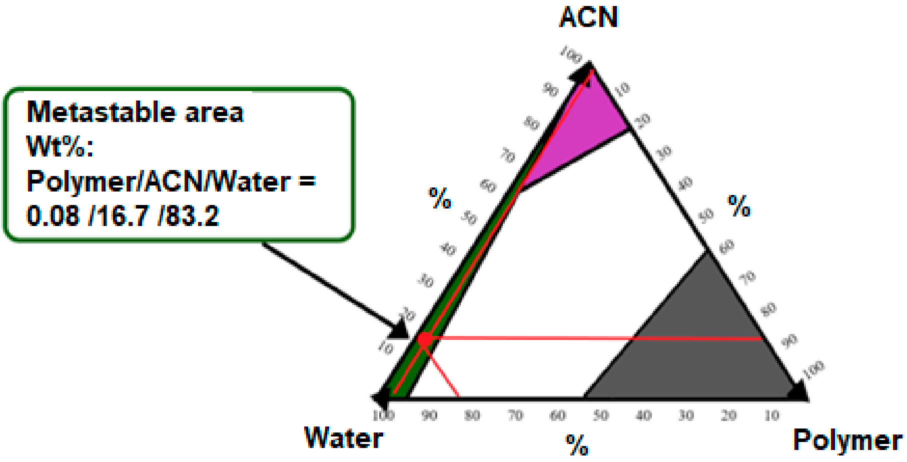

Figure S4. Three-component phase diagram polymer/organic solvent/water with selected optimal wt% of components to prepare nanoparticles up to 100 nm in diameter.

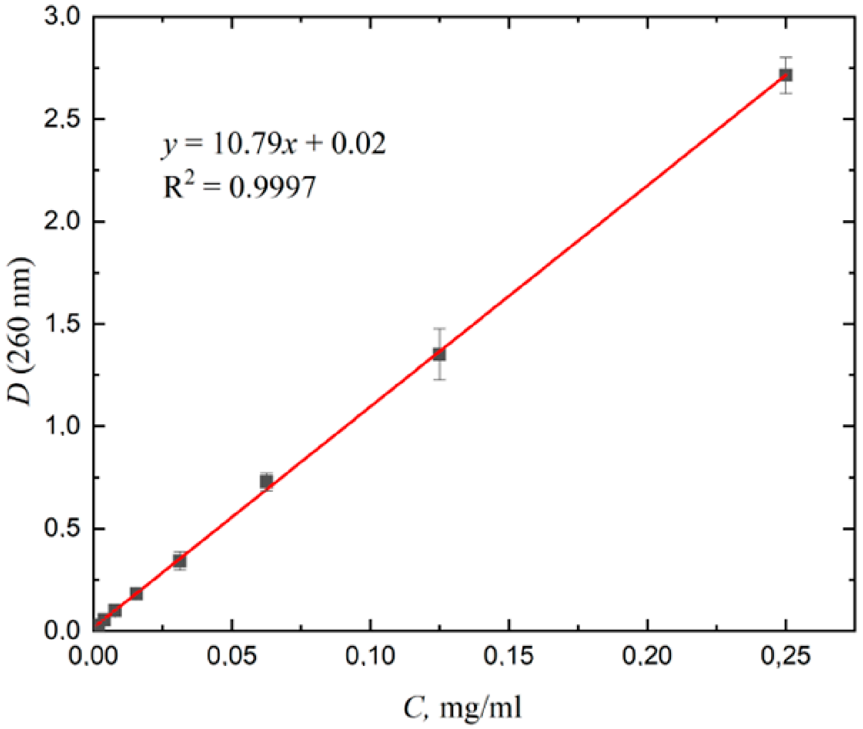

Figure S5. Calibration plot of absorbance on the different concentration of dioxadet solutions (in dichloromethane).

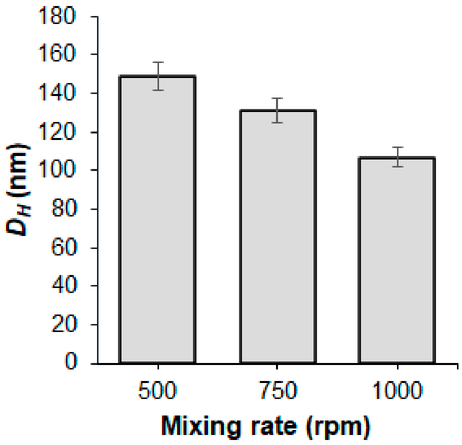

Figure S6. Dependence of the hydrodynamic diameter of mPEG-*b*-PLA nanoparticles on the mixing rate of the organic (ACN/THF) and aqueous (deionized water) phases.

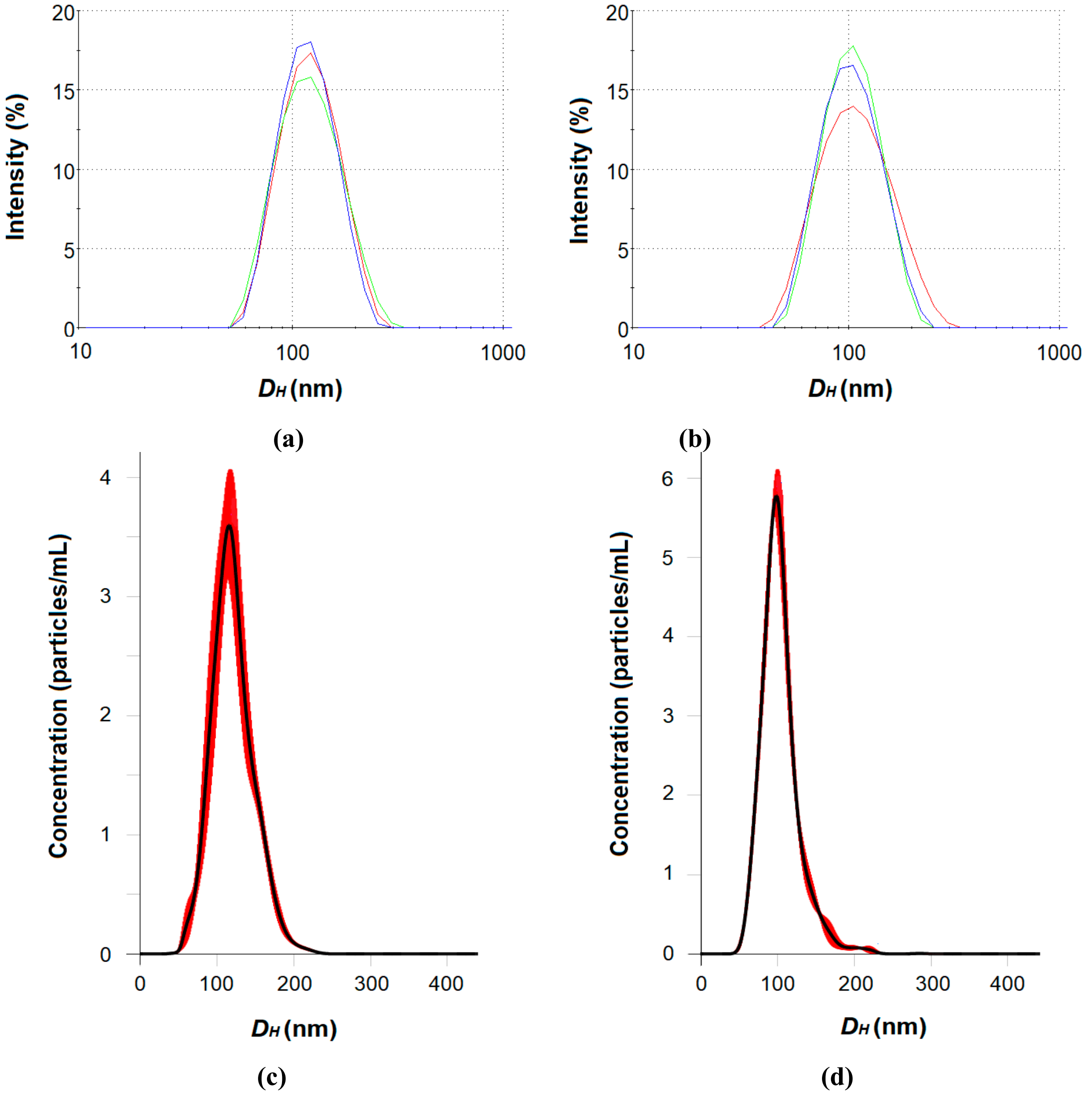

Figure S7. Distribution of the mPEG-*b*-PLA (**a**,**c**) and mPEG-*b*-PCL (**b**,**d**) nanoparticles by hydrodynamic diameter analyzed by DLS (**a**, **b**), and NTA (**c**, **d**).

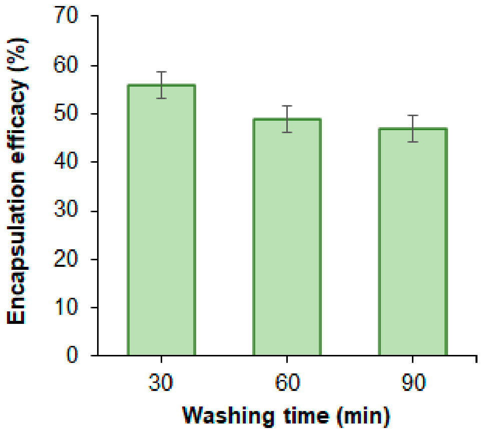

Figure S8. Dependence of the purification time of the prepared nanoformulations on encapsulation efficacy.

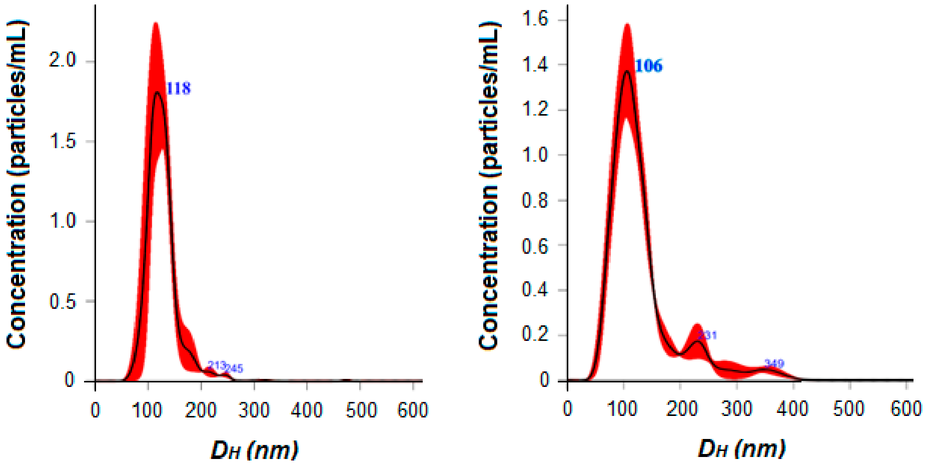

(a) **(b)**

Figure S9. Distribution in hydrodynamic diameter of the nanomedicines (NTA; samples prepared at the initial DOD amount equal to 50 µg/mg polymer: (**a**) DOD/mPEG-*b*-PLA; (**b**) DOD/mPEG-*b*-PCL; the red line represents the average of three measurements).

(a)

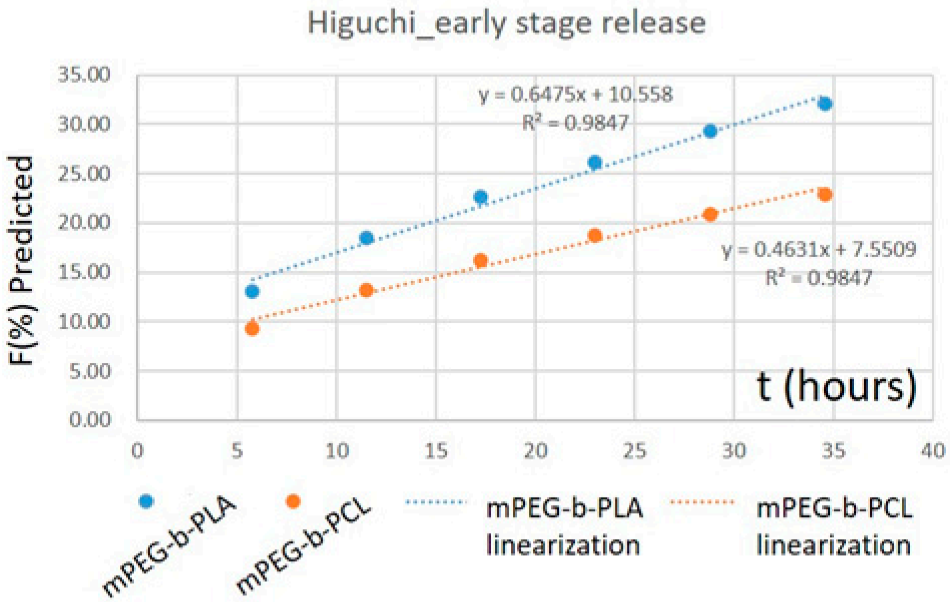

(b)

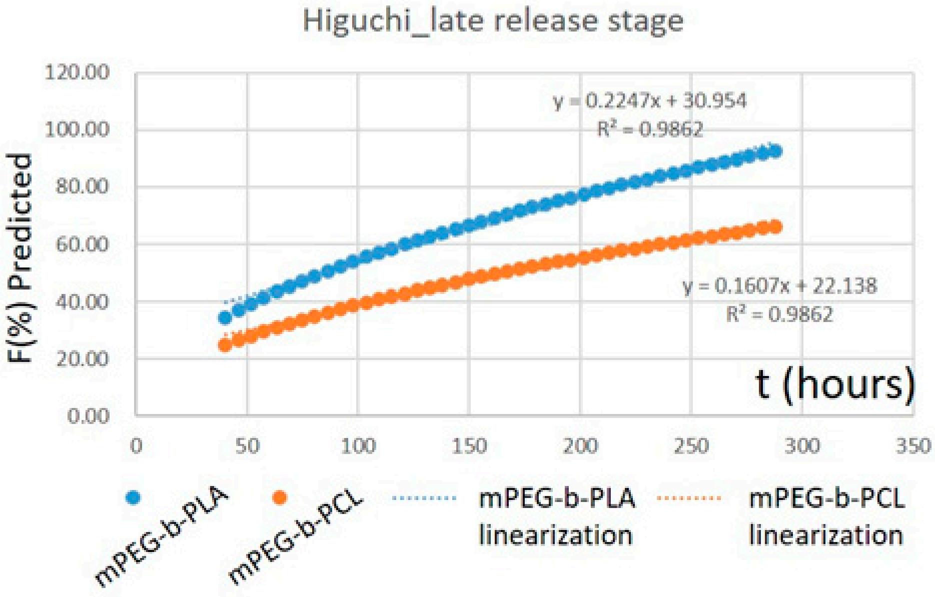

Figure S10. Linearization of early and late DOD release from mPEG-*b*-PLA (**a**) and mPEG-*b*-PCL (**b**) as predicted with Higuchi model. Quite clear linearizations show two stages with two different diffusion coefficients.

(a)

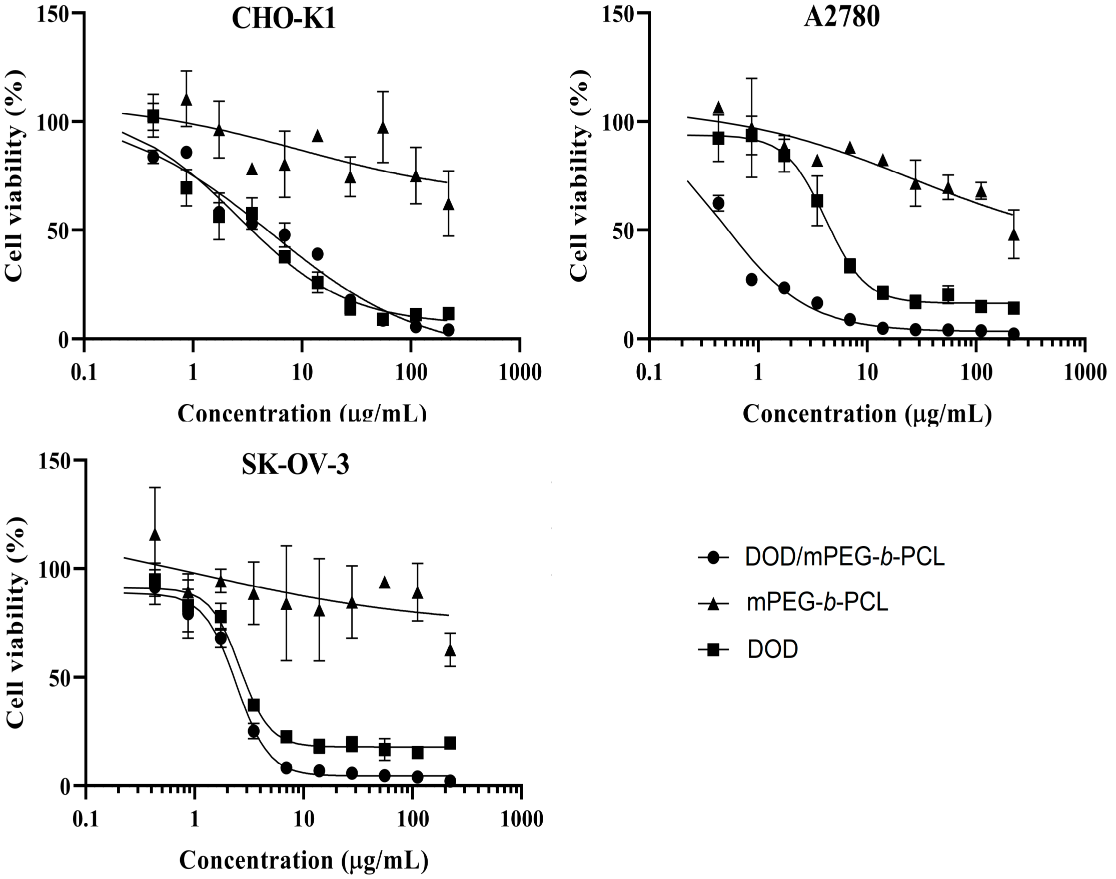

CHO-K1
A2780
SK-OV-3
Cell viability (%)
Concentration (μg/mL)
DOD/mPEG-b-PCL
mPEG-b-PCL
DOD

(b)

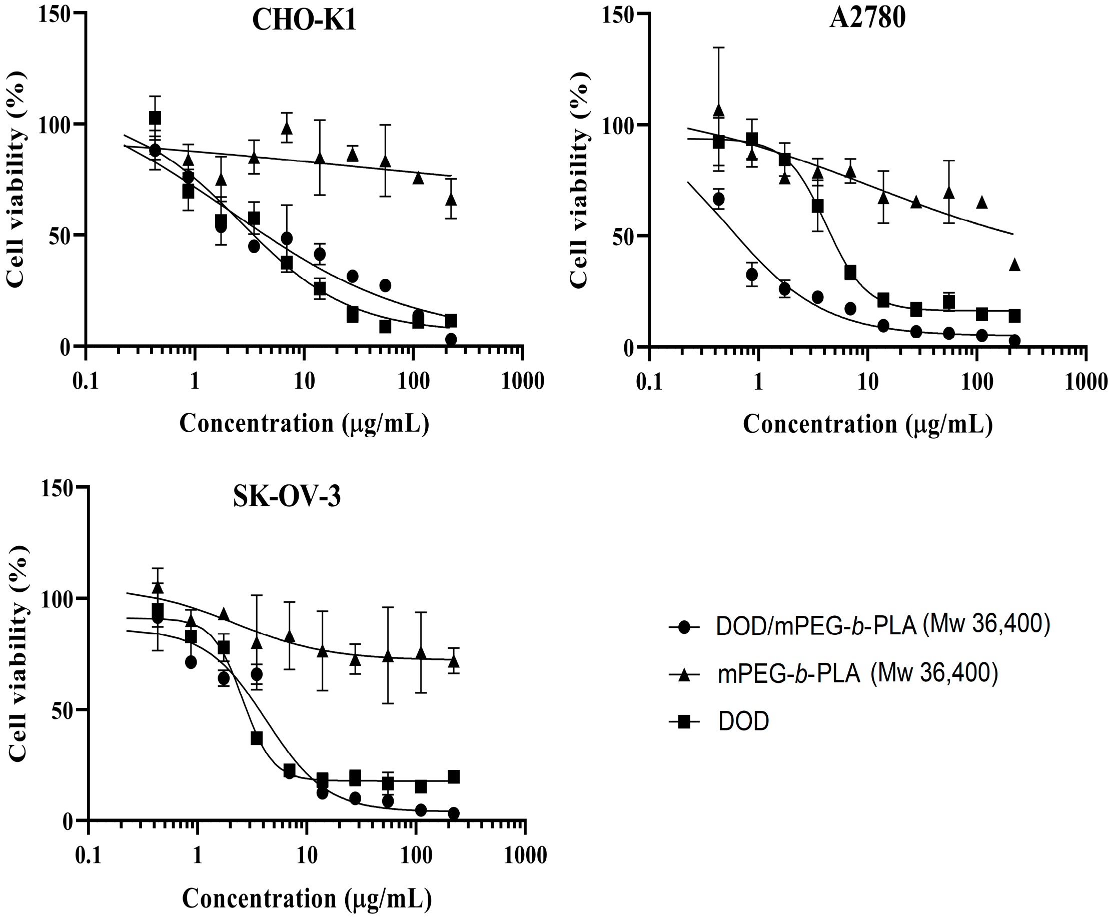

CHO-K1
A2780
SK-OV-3
Cell viability (%)
Concentration (μg/mL)
DOD/mPEG-b-PLA (Mw 36,400)
mPEG-b-PLA (Mw 36,400)
DOD

(c)

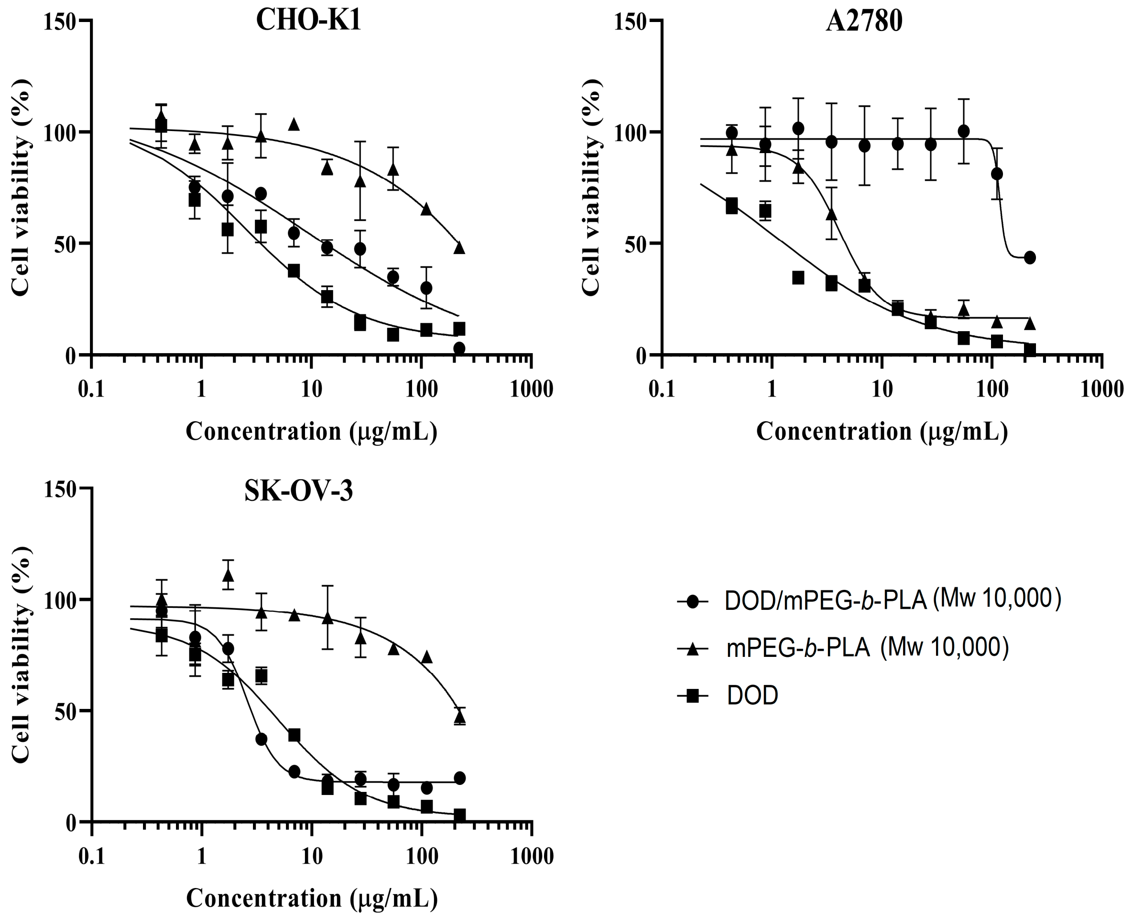

Figure S11. Dependence of cell viability on the concentration of nanomedicines, empty polymer nanoparticles and free dioxadet for different cell lines: **(a)** a series for mPEG-*b*-PCL; **(b)** a series for mPEG-*b*-PLA (M_w 36,400) and **(c)** a series for mPEG-*b*-PLA (M_w 10,000).

Table S1. Dependence of the yield of nanoparticles based on mPEG-*b*-PLA (sample #7, Table 1) and mPEG-*b*-PCL (sample #11, Table 1) (nanoprecipitation from organic phase to water).

Initial drug amount (µg/mg of polymer)	Yield (%)
**mPEG-*b*-PLA**	
50	74
100	72
200	75
300	75
400	72
500	70
**mPEG-*b*-PCL**	
50	44
100	46
200	46
300	45
400	46
500	43
